# Assessment of hepatocellular carcinoma treatment response with LI-RADS: a pictorial review

**DOI:** 10.1186/s13244-019-0801-z

**Published:** 2019-12-18

**Authors:** Nicolas Voizard, Milena Cerny, Anis Assad, Jean-Sébastien Billiard, Damien Olivié, Pierre Perreault, Ania Kielar, Richard K. G. Do, Takeshi Yokoo, Claude B. Sirlin, An Tang

**Affiliations:** 1 Department of Radiology, Centre hospitalier de l’Université de Montréal (CHUM), Montreal, Québec Canada; 2 Centre de recherche du Centre hospitalier de l’Université de Montréal (CRCHUM), Montréal, Québec Canada; 3 Joint Department of Medical Imaging, University of Toronto, Toronto, Canada; 4 Department of Radiology, Memorial Sloan Kettering Cancer Center, New York, NY USA; 5 Department of Radiology, University of Texas Southwestern Medical Center, Dallas, TX USA; 6 Advanced Imaging Research Center, University of Texas Southwestern Medical Center, Dallas, TX USA; 7 Liver Imaging Group, Department of Radiology, University of California San Diego, San Diego, CA USA; 8 Department of Radiology, Radio-oncology and Nuclear Medicine, University of Montreal, Montreal, Canada

**Keywords:** LIRADS, LI-RADS Treatment Response, Hepatocellular carcinoma, Magnetic resonance imaging, Computed tomography, Locoregional

## Abstract

Computed tomography (CT) and magnetic resonance imaging (MRI) play critical roles for assessing treatment response of hepatocellular carcinoma (HCC) after locoregional therapy. Interpretation is challenging because posttreatment imaging findings depend on the type of treatment, magnitude of treatment response, time interval after treatment, and other factors. To help radiologists interpret and report treatment response in a clear, simple, and standardized manner, the Liver Imaging Reporting and Data System (LI-RADS) has developed a Treatment Response (LR-TR) algorithm. Introduced in 2017, the system provides criteria to categorize response of HCC to locoregional treatment (e.g., chemical ablation, energy-based ablation, transcatheter therapy, and radiation therapy). LR-TR categories include Nonevaluable, Nonviable, Equivocal, and Viable. LR-TR does not apply to patients on systemic therapies. This article reviews the LR-TR algorithm; discusses locoregional therapies for HCC, treatment concepts, and expected posttreatment findings; and illustrates LI-RADS treatment response assessment with CT and MRI.

## Key points

- Liver Imaging Reporting and Data System (LI-RADS) Treatment Response (LR-TR) categories are Nonevaluable, Nonviable, Equivocal, and Viable.
- Hepatocellular carcinoma (HCC) locoregional therapies can be broadly divided into locoablative therapy, transcatheter therapy, and radiation therapy.
- Nodular arterial phase hyperenhancement (APHE) or washout appearance along the margin of a treated observation indicates recurrence or residual viable tumor.
- After radiation-based treatments, intralesional enhancement and washout appearance may persist for months but eventually regress.
- Since multimodal therapy is often used to treat HCC, knowledge of prior therapy and review of prior imaging facilitate proper assessment of treatment response.

## Introduction

Hepatocellular carcinoma (HCC) is worldwide the sixth most common cancer overall and fourth most common cause of cancer-related mortality [1]. Several therapies have been developed for treating patients with this aggressive malignancy. Broadly divided into surgery, locoablative therapy, transcatheter therapy, radiation therapy, and systemic therapy, these therapies can be used alone or in combination; planned in a single or in multiple sessions; and performed with curative, downstaging, bridge, debulking, or palliative intent [2, 3].

Assessing treatment response after therapy is essential for determining prognosis and informing future management. Treatment response assessment is based largely on imaging with computed tomography (CT) or magnetic resonance imaging (MRI). Interpretation of liver imaging following HCC treatment can be challenging because findings depend on the type(s) of therapy, the number of rounds of therapy, the magnitude of treatment response, the timing of imaging after therapy, and the cumulative effect of therapy on underlying liver function. Compounding the diagnostic difficulty is that most patients with HCC have underlying cirrhosis with parenchymal and perfusional heterogeneity and are at high risk for developing new HCCs elsewhere in the liver.

To address these challenges, the Liver Imaging Reporting and Data System (LI-RADS) introduced a Treatment Response (LR-TR) algorithm in 2017 to categorize response of HCC to locoregional therapy (e.g., locoablative, transcatheter, radiation). Endorsed by the American College of Radiology, the LR-TR algorithm was modeled after and designed to complement the LI-RADS Diagnostic Algorithms, which assign categories to imaging-detected untreated liver observations reflecting their perceived probability of HCC [4]. Analogously, the LR-TR algorithm assigns response categories to treated liver observations reflecting their perceived probability of viability or recurrence after locoregional therapy. The algorithm does not apply to systemic therapies and should be applied with caution after surgical resection.

This article reviews the LR-TR algorithm; discusses locoregional therapies for HCC, treatment concepts, and expected posttreatment findings; and illustrates treatment response assessment with CT and MRI. Despite the numerous treatments and variability in posttreatment findings, LI-RADS provides a simple and practical way to assess and report treatment response.

## Assessment of treatment response

CT and MRI are usually performed at regular intervals after locoregional therapy to assess treatment response. The goal of posttreatment imaging is to recognize residual or recurrent tumor requiring further treatment, identify complications of therapy, and detect and characterize new or additional observations elsewhere in the liver. A crucial first step in applying the LR-TR algorithm is to evaluate the adequacy of imaging technique. Radiologists should be aware that multiphase contrast-enhanced imaging—including a late arterial phase and at least two post-arterial phases (e.g., portal venous, delayed venous)—is required. Further, although often not needed to characterize lesions before treatment, a precontrast CT of the liver is recommended for patients after locoregional therapy to facilitate differentiation of posttreatment changes from abnormal enhancement of viable tumor. The detailed technical requirements for posttreatment CT and MRI imaging are provided in LI-RADS v2018 manual [5].

## LR-TR response categories

**Fig. 1:**
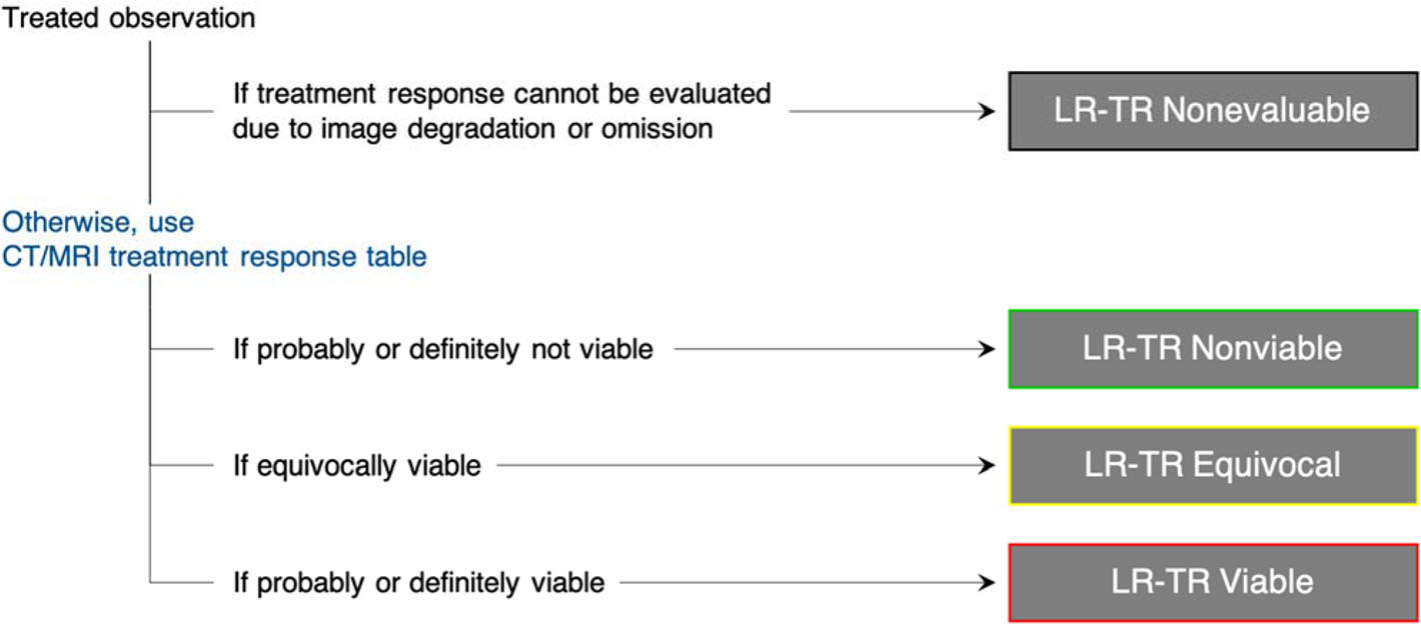
LI-RADS CT/MRI Treatment Response algorithm. Reprinted, with permission, from ACR [5]

**Fig. 2:**
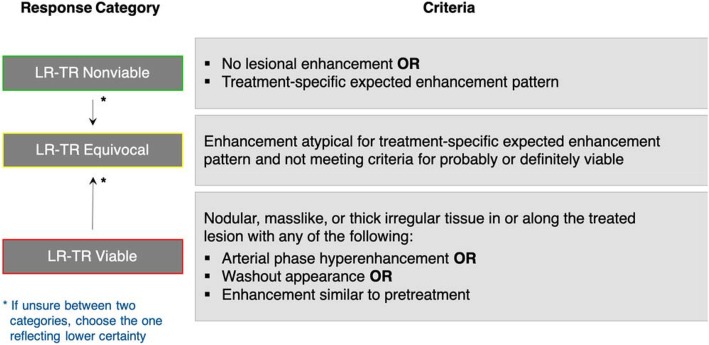
LI-RADS CT/MRI Treatment Response criteria and tiebreaking rule. Diagram created by authors. Adapted and reprinted, with permission, from ACR [5]

When reporting a liver observation (i.e., a distinctive area compared to background liver) in a treated patient, the LR-TR algorithm (Fig. 1) should be followed and one of the four LR-TR categories (Fig. 2) below should be assigned based on the enhancement features listed in Table 1. Untreated observations elsewhere in the liver should be assigned a LI-RADS CT/MRI diagnostic category [4].

**Table 1:** Treatment response features. Reprinted, with permission, from ACR [5]

Features	Definitions
Viability	Presence of live tumor cells within or along the margin of a treated lesion. Radiologic viability is not synonymous with pathologic viability as imaging is not sensitive to microscopic or small foci of residual tumor.
Treatment-specific expected enhancement	Expected temporal and spatial pattern of posttreatment enhancement attributable to treatment-related changes in parenchymal perfusion. Posttreatment enhancement patterns may not reliably differentiate viable from nonviable tumor. The most appropriate response category may be LR-TR Equivocal.
No lesional enhancement	Absence of enhancement within or along the margin of a treated lesion. Note: complete disappearance after locoregional treatment is considered equivalent to absence of enhancement.
Posttreatment APHE	Nodular, masslike, or thick and irregular arterial phase hyperenhancement (APHE) contained within or along the margin of a treated lesion suggests posttreatment tumor viability.
Posttreatment “washout”	Nodular, masslike, or thick and irregular washout appearance contained within or along the margin of a treated lesion suggests posttreatment tumor viability.
Posttreatment enhancement similar to pretreatment	Nodular, masslike, or thick and irregular enhancement similar to pretreatment enhancement in all postcontrast phases contained within or along the margin of a treated lesion suggests posttreatment tumor viability, even in the absence of APHE or washout appearance.

### LR-TR Nonevaluable

This category is assigned when the treatment response cannot be evaluated due to poor image quality or inadequate technique (e.g., failure to obtain the required phases).

### LR-TR Nonviable

The nonviable category should be assigned to treated lesions with no perceived enhancement or demonstrating only expected posttreatment enhancement patterns. The latter may depend on the applied locoregional treatment used and the time interval after treatment.

### LR-TR Equivocal

This category is applied to treated observations that cannot be confidently categorized as viable or nonviable due to overlapping enhancement features in the absence of technical or patient-related limitations.

### LR-TR Viable

The viable category should be assigned to treated observations with nodular, masslike, or thick irregular regions of arterial phase hyperenhancement (APHE), washout appearance, or enhancement similar to pretreatment tumor. These features indicate the presence of viable tumor cells with high certainty. This is valid for all locoregional treatments with the exception of radiation therapy as discussed further.

It is important to note that these LR-TR categories are assigned based on perceived probabilities of macroscopic tumor viability at imaging. They are not intended to reflect microscopic tumor viability visible only at histology. Thus, LR-TR Nonviable means there is no imaging evidence of gross viable tumor, but it does not exclude the presence of viable tumor cells visible microscopically on histology slides obtained from tissue sampling.

## Locoregional therapies for HCC

**Fig. 3:**
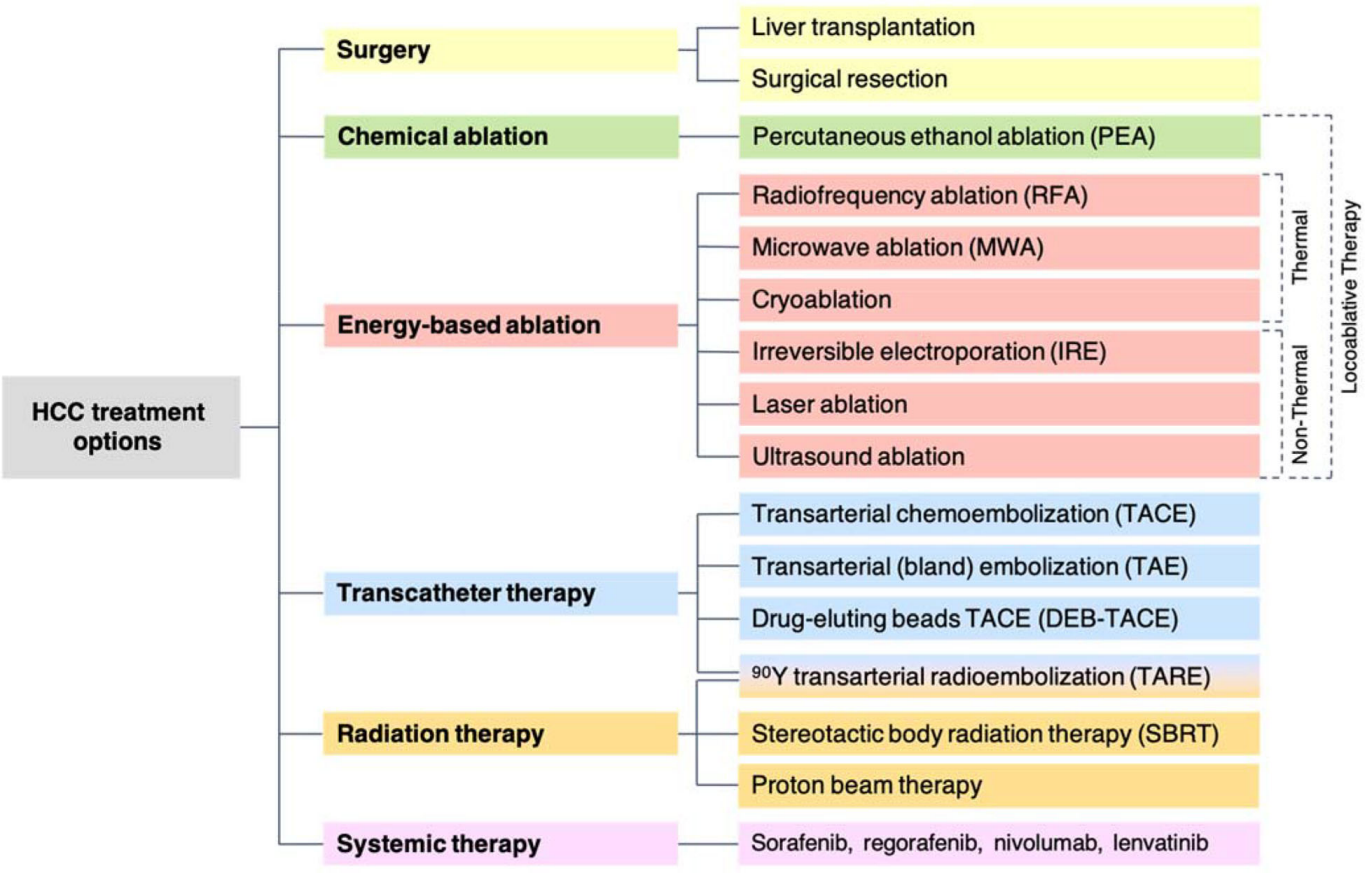
Hepatocellular carcinoma (HCC) treatment options divided by modality. Locoablative therapies (PEA, RFA, MWA), transcatheter therapies (TAE, TACE, DEB-TACE, TARE) and radiation therapies (SBRT) are the most commonly used locoregional treatments for HCC treatment and are reviewed in this article

Therapeutic options for HCC include surgery (liver transplantation or resection), locoregional therapies, and systemic therapy (e.g., sorafenib, regorafenib, nivolumab, lenvatinib) (Fig. 3) [6, 7]. In general, surgery and locoablative therapies are performed with curative intent, while transcatheter, radiation, and systemic therapies are applied mainly for downstaging, bridging, debulking, or palliation. Therapies can be classified by their intent:

- *Curative therapy* refers to treatment intended to completely eliminate HCC.
- *Downstaging therapy* refers to treatment intended to reduce the tumor stage from beyond to within accepted transplant criteria and allow transplantation.
- *Bridging therapy* refers to treatment intended to maintain patients already within accepted transplant criteria and to prevent dropout due to tumor progression.
- *Debulking therapy* refers to treatment of advanced HCC intended to prolong survival despite massive, multifocal, or infiltrative disease ineligible for above treatments.
- *Palliative therapy* refers to treatment intended to alleviate symptoms and improve quality of life rather than reducing or eliminating tumor burden.

Locoregional therapies, the topic of this review article, are classified by LI-RADS as summarized below and in Fig. 3 [6, 7].

- *Locoablative therapies:* These include chemical ablation (percutaneous ethanol ablation [PEA]) and energy-based ablation. The latter is divided into thermal ablation (radiofrequency ablation [RFA], microwave ablation [MWA], cryoablation) and non-thermal ablation (irreversible electroporation [IRE], laser ablation, ultrasound ablation).
- *Transcatheter therapies:* These include transarterial embolization (transarterial bland embolization [TAE], transarterial chemoembolization [TACE], drug-eluting beads TACE [DEB-TACE], ^90^Y transarterial radioembolization [TARE]).
- *Radiation therapies:* These include stereotactic body radiation therapy (SBRT) and proton beam therapy.

For each locoregional therapy, we will provide an overview of the treatment procedure, its mechanism of action, and its complications; summarize its indications and reported efficacy; describe the expected treatment response; and illustrate the LR-TR nonviable, equivocal, and viable categories at CT and MRI. Emphasis is placed on key concepts that help radiologists understand the expected post therapy findings and their evolution over time. Cryoablation, non-thermal ablation, and proton beam therapy are not commonly used for HCC and are not further discussed.

## Locoablative therapies

### Technique, mechanism of action, and complications

The most common locoablative therapies for HCC are percutaneous ethanol ablation (PEA), radiofrequency ablation (RFA), and microwave ablation (MWA).

**Fig. 4:**
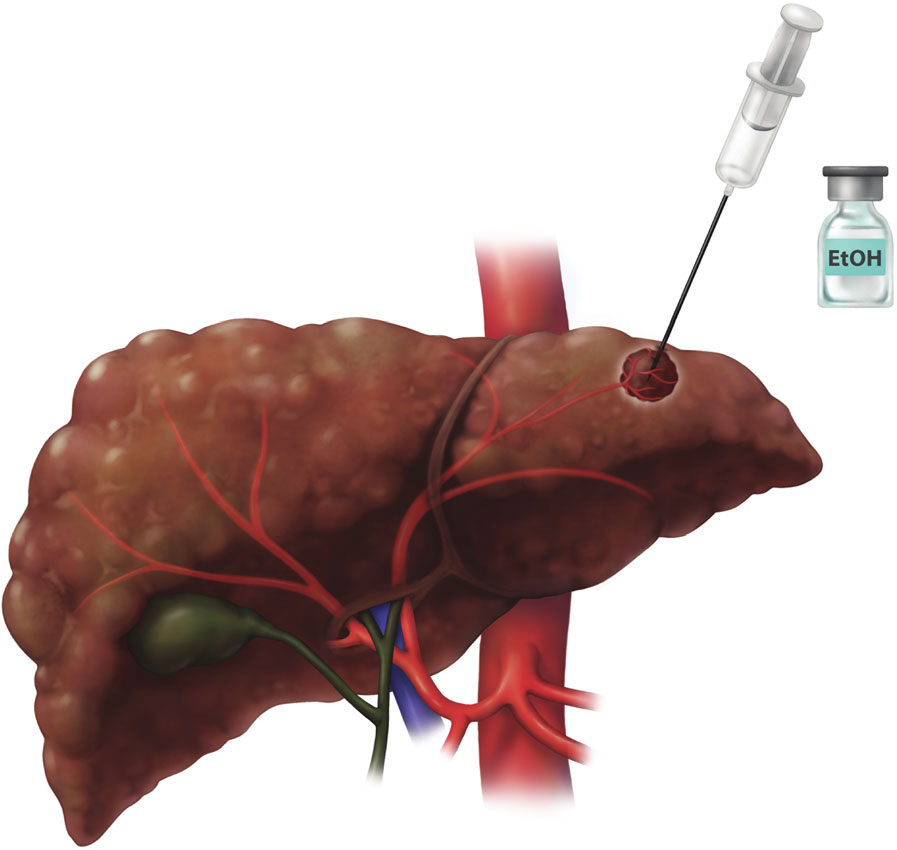
Percutaneous ethanol ablation (PEA). Absolute ethanol is injected in the tumor. Multiple sessions and prolonged treatment time may be required

Percutaneous ethanol ablation, a chemical ablation procedure, was the first locoablative therapy used clinically for treating early-stage HCC. In this technique, one or more needles are inserted percutaneously into the tumor under image guidance (Fig. 4). Absolute ethanol is injected into the tumor, in a volume ranging from 1 to 10 mL depending on the tumor size. Ethanol is cytotoxic and upon entering the tumor microcirculation causes coagulation necrosis via platelet aggregation, small vessel thrombosis, and ischemia [8]. With PEA, tumor targeting may be difficult as the ablated tumor margins are less clearly identified during the procedure [9]. To overcome this issue, two treatment sessions per week for up to 12 sessions may be required to induce complete tumor necrosis [10, 11].

RFA and MWA are more recently developed therapies. As opposed to the cytotoxic effect of PEA, RFA and MWA rely on heat to damage and to kill cells. Heat-induced damage and death of HCC and surrounding hepatocytes is proportional to temperature and exposure time [12]. Cellular damage occurs in 60 min at 46 °C, while cell death occurs in 4–6 min at 50–52 °C and is nearly instantaneous between 60 and 100 °C [13]. Coagulative necrosis is the main cell death mechanism. However, when the delivered energy is insufficient, for example, towards the periphery of the ablation zone, a transition is observed from necrosis to apoptosis [14]. The energy is deposited into the tissue using percutaneously inserted needle-like elements known as “applicators” [6]. Based on the physical principles behind each technique, the applicator in RFA is also termed an electrode while the applicator in MWA is also termed an antenna. The applicators are inserted percutaneously under imaging guidance; they can be straight or expandable, and they can be used alone or in clusters.

**Fig. 5:**
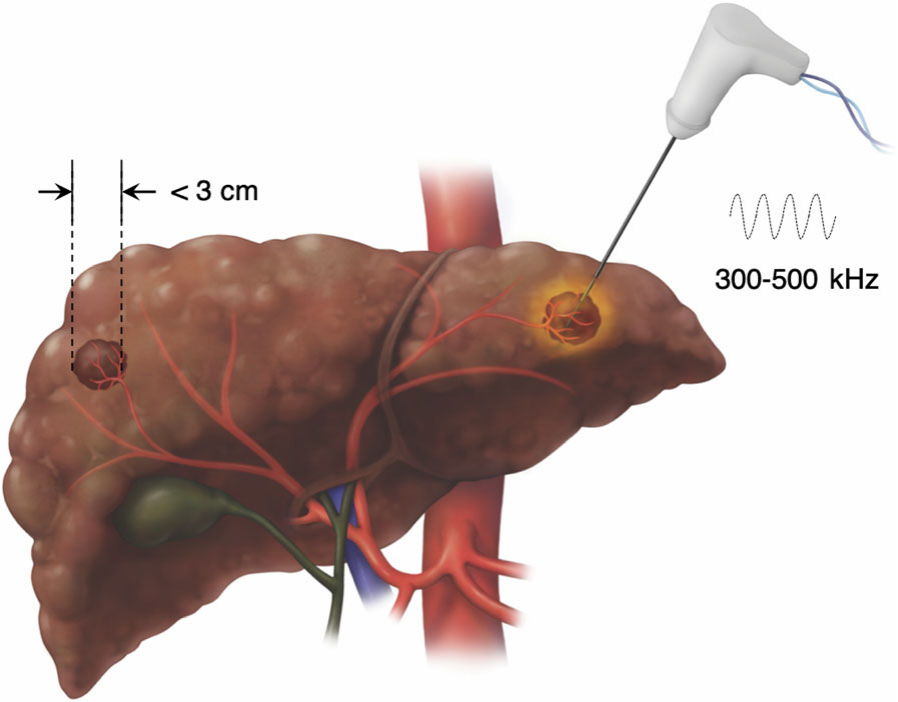
Radiofrequency ablation (RFA). Treatment is typically used to treat hepatocellular carcinomas under 3 cm in a non-perivascular location

RFA is currently indicated to treat tumors up to about 3 cm in size (Fig. 5). To achieve proper energy delivery and tumoral ablation, one to three applicators are advanced through the tumor and at least 5 mm beyond its deepest margin. Alternating current within the radiofrequency spectrum (between 300 and 500 kHz) is delivered to a closed-circuit between two electrodes: the applicator in the tumor and a grounding pad positioned on the skin far from the ablation site (e.g., on the thighs). The resistive effect around the noninsulated applicator tip heats the tissue in a centrifugal direction [13]. Temperatures as high as 105 °C can be achieved, which boils, vaporizes, necroses, and chars the tissue [13]. The charring has the unintended consequence of increasing tissue impedance, which limits the transmission of energy to adjacent cells thus reducing RFA efficacy. Some internally cooled applicators alternate between periods of high and low energy deposition which partially offsets the impedance effect [15]. Another limiting factor of RFA is the “heat sink effect” that reduces treatment efficacy for perivascular tumors due to heat loss into large vessels [16, 17]. For tumors adjacent to large vessels alternative therapies may be preferable. Procedure time generally ranges from 10 to 16 min per tumor depending on its size.

**Fig. 6:**
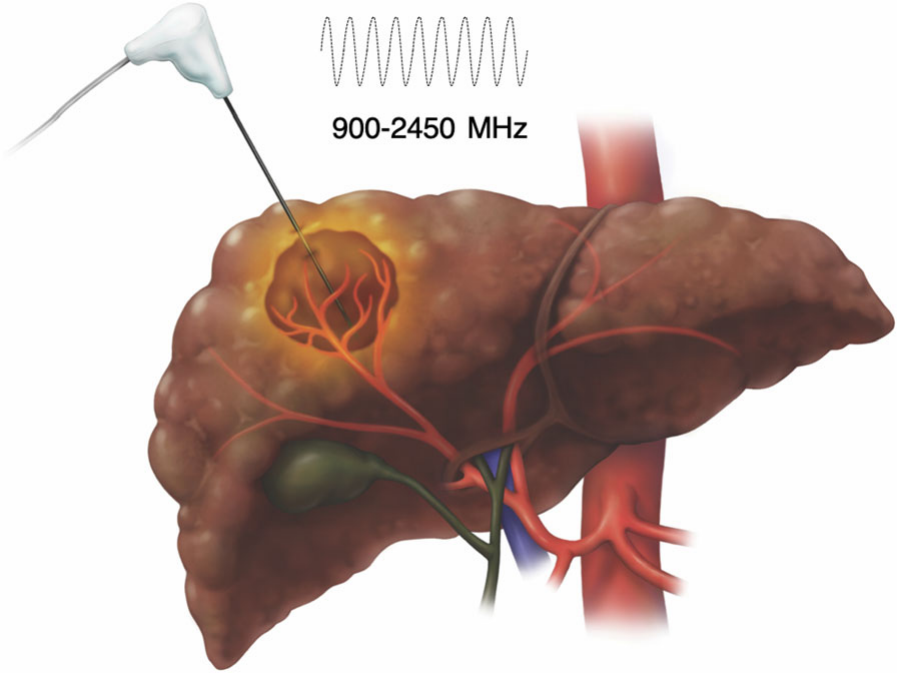
Microwave ablation (MWA). Compared to radiofrequency ablation, larger tumors can be targeted in shorter treatment duration even in perivascular locations

MWA is similar to RFA as one or more needle-like applicators are inserted percutaneously into the tumor using image guidance (Fig. 6). Grounding pads are unnecessary, which simplifies the setup and eliminate the risk of grounding pad burns. MWA is achieved by generating a rapidly oscillating magnetic field around the applicator (antenna) at 900 MHz to 2450 MHz, frequencies 3000 to 5000 times greater than RFA [18]. Unlike RFA where resistive forces are predominant, friction is the main energy delivery mechanism in MWA. The oscillating electromagnetic field causes water and other polar molecules to flip billions of time per seconds, which heats tissues around the noninsulated tip of the applicator [18]. The electromagnetic field created by MWA is not affected by tissue boiling and charring and the deposited energy is too intense to be dissipated by flowing blood [19]. Additionally, simultaneous multi-probe ablation act synergistically to create a larger ablation zone than sequential single-probe ablation [19]. For these reasons, MWA achieves higher intratumoral temperatures faster, more uniformly, and over a larger volume than RFA; takes less time to perform (under 10 min per tumor); and can be used to treat larger tumors as well as tumors adjacent to large vessels.

Ablative therapies can damage blood vessels and bile ducts, potentially resulting in hematomas and bilomas within or immediately adjacent to the ablation zone. Small arterioportal shunts may be induced by the procedure in up to 25% of cases; the vast majority heal spontaneously at 1–4 months follow-up [17, 20]. Additionally, the parenchyma along the ablation margin and in the surrounding liver may become hyperemic; the hyperemia typically resolves after about 6 months. Complications post locoablative therapies are infrequent (2–3%) and are similar for PEA, RFA, and MWA: hemorrhage, infection, abscess, visceral organ injury, bile leak, liver failure, portal vein thrombosis, cardiac arrythmias, and pneumothorax [17, 20]. Risk of tumor seeding (0.5–3%) can be reduced during RFA and MWA by cauterization of the needle trajectory at the time of probe removal and by avoiding direct puncture of subcapsular tumors [21]. Serious skin burns (0.2%) at the grounding pad site are an uncommon and unique complication associated with RFA; these have become rare with technical advancements [22].

### Indications and efficacy

Although PEA is simple, safe, and inexpensive, it has a practical drawback due to multiple treatment sessions and a prolonged treatment time [23]. Randomized controlled trials have shown the superiority of RFA compared to PEA in local control and tumor recurrence [10, 11, 24, 25]. PEA is typically used when thermal methods are at higher risks of complication or reduced efficacy due to tumor proximity to major bile ducts, large vessels, liver capsule, abdominal organs, and heart.

RFA is considered the first line of treatment in non-surgical patients for HCC under 3 cm [26–28]. Randomized controlled trials have shown inconsistent results comparing long-term survival between patients undergoing RFA versus hepatic resection [29]. However, RFA is less invasive, has fewer complications, has less impact on hepatic reserve, requires shorter hospitalization, and is less expensive than surgery [26–28]. While RFA remains the standard of care for locoregional therapy of non-surgical patients and early-stage HCC [30, 31], MWA shows promising results for local control and survival and has already been implemented in many institutions. No published study has confirmed superiority of MWA to RFA: both techniques have been shown to have low recurrence rate at 2 years in a recent randomized controlled trial for up to three tumors < 4 cm in size [32].

### Expected treatment response

Posttreament appearance after PEA, RFA, and MWA is similar but non-identical and depends on the time interval after therapy. At first posttreament follow-up, typically 1–3 months, the ablation zone should encompass the entire tumor volume and as well as the surrounding hepatic parenchyma along the tumor margin. The anticipated diameter of the ablation zone for RFA and MWA is typically 5 to 10 mm greater than the treated tumor [33]. With PEA, the ablation zone volume is harder to predict; margins may be the same size or larger than the tumor. Attenuation and signal intensity of the ablation zone are variable. Heterogeneous hyperdensity/intensity on unenhanced CT or T1-weighted MRI can be seen, reflecting coagulative necrosis. The ablation zone is typically hypointense on T2-weighted MRI except for portions liquefied by necrosis or containing fluid collections (bilomas, hematomas). With RFA and MWA, due to tract cauterization, a needle trajectory in the hepatic parenchyma towards the tumor is often seen. The ablation zone may contain gas foci for up to a month posttreatment. In the majority of asymptomatic patients, these reflect air introduced by the needles during the procedure or tissue necrosis rather than infection [34, 35].

The attenuation and intensity of viable tumor may be indistinguishable from successfully ablated tissue, and contrast-enhanced imaging is essential to evaluate viability or recurrence [36]. Uniform thin peripheral enhancement along ablation margin and a smooth rind of parenchymal enhancement around ablation zone may be present in the first 6 months due to reactive hyperemia [8]. By 6 months, the ablation zone begins to involute, although a residual ablation zone can persist indefinitely, and marginal enhancement begins to resolve. Wedge-shaped areas of transient hepatic attenuation/intensity difference can be seen adjacent to the ablation zone due to posttreatment arterioportal shunts especially in the first 4 months. These perfusion variants can be distinguished from residual viable tumor by their geographic shape and absence of mass effect, washout appearance, or capsule appearance.

At any point posttreatment, presence of nodular APHE, washout appearance, or enhancement similar to pretreatment suggests recurrence or residual viable tumor.

The expected treatment response appearances after PEA, RFA, and MWA are summarized in Fig. 7. LR-TR nonviable, equivocal, and viable representative CT and MRI cases after PEA (Figs. 8, 9, and 10), RFA (Figs. 11, 12, and 13), and MWA (Figs. 14, 15, and 16) are provided.

**Fig. 7:**
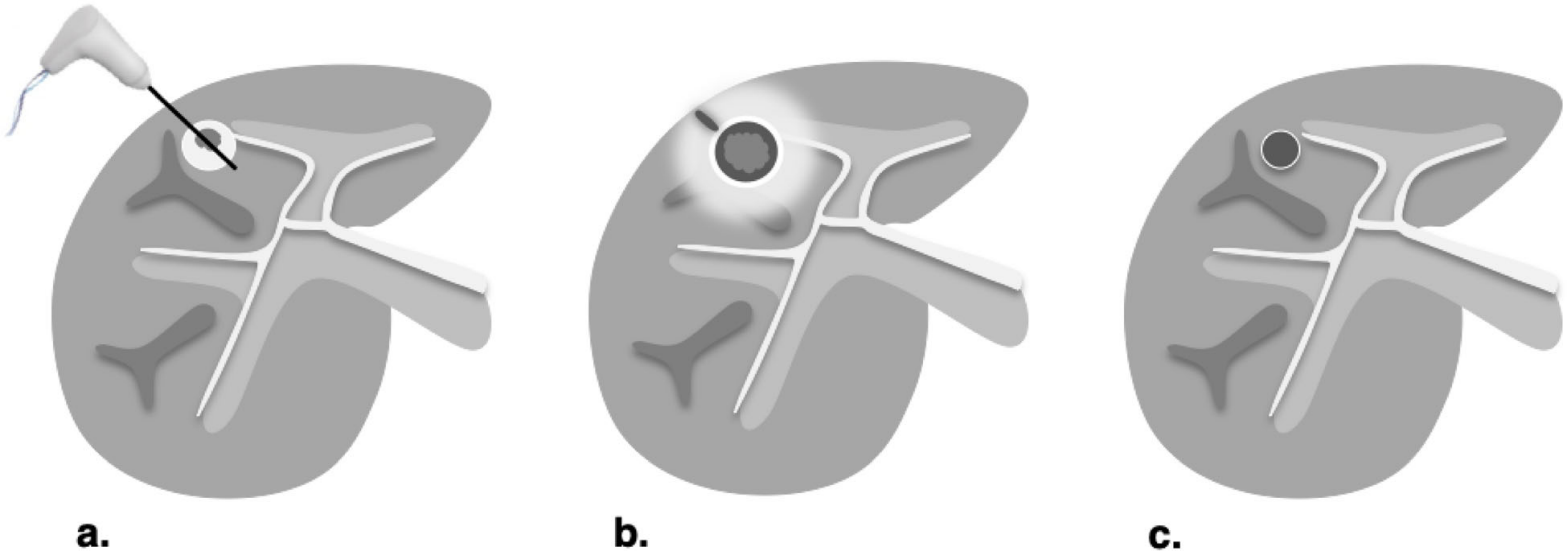
Expected treatment response after percutaneous ethanol ablation (PEA), radiofrequency ablation (RFA) and microwave ablation (MWA). Axial contrast-enhanced CT images of the liver obtained in late arterial phase are illustrated: **a** Pretreatment: RFA is used with curative intent of early-stage hepatocellular carcinoma (≤ 3 cm) in non-surgical patients. PEA is typically used when RFA is unsafe or contraindicated. MWA may target larger tumor with curative intent but additional studies are needed. Larger tumor (> 3 cm) may be targeted for downstaging purpose or as a bridging therapy prior to transplantation alone or in combination with other treatments. **b** 1–3 months posttreatment: Diameter of ablation zone at the time of treatment is usually 5 to 10 mm greater than the treated lesion. The following features may be seen: intratumoral gas foci up to 1 month posttreatment, thin linear peripheral enhancement along ablation zone, smooth rind or wedge-shaped parenchymal enhancement around ablation zone, intralesional hyperdensity/intensity on unenhanced CT or on T1-weighted MRI (reflecting coagulation necrosis), and hypodense liver parenchyma may be seen along needle trajectory. **c** ≥ 6 months posttreatment: ablation zone involutes over time. Thin linear peripheral enhancement along ablation zone decreases. Parenchymal enhancement resolves. At any point posttreatment, presence of nodular arterial phase hyperenhancement, washout appearance, or enhancement similar to pretreatment indicates recurrence or residual viable tumor

**Fig. 8:**
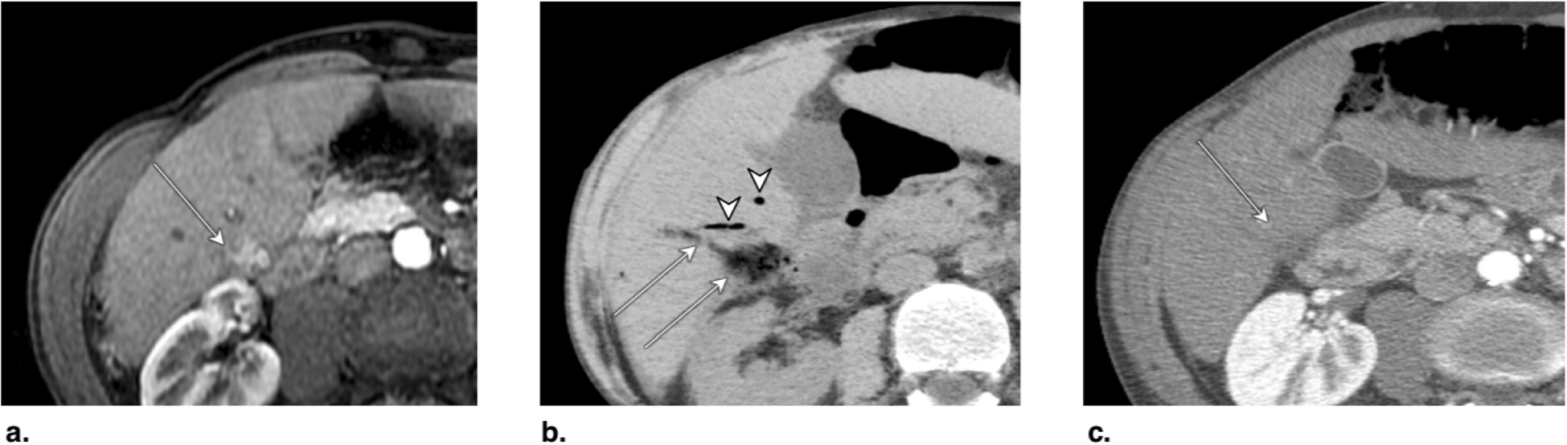
**a** Axial T1-weighted MR in late arterial phase (AP) obtained pretreatment: image shows nonrim arterial phase hyperenhancement of a 2-cm LR-4 observation (arrow). **b** Axial-unenhanced CT MinIP obtained immediately post percutaneous ethanol ablation (PEA): image shows pneumobilia (arrowheads), ablation cavity, and needle trajectory (arrows). **c** Axial CT in late AP obtained 4 months post PEA: image shows no enhancement and cavity retraction (arrow). The treated observation is categorized LR-TR Nonviable

**Fig. 9:**
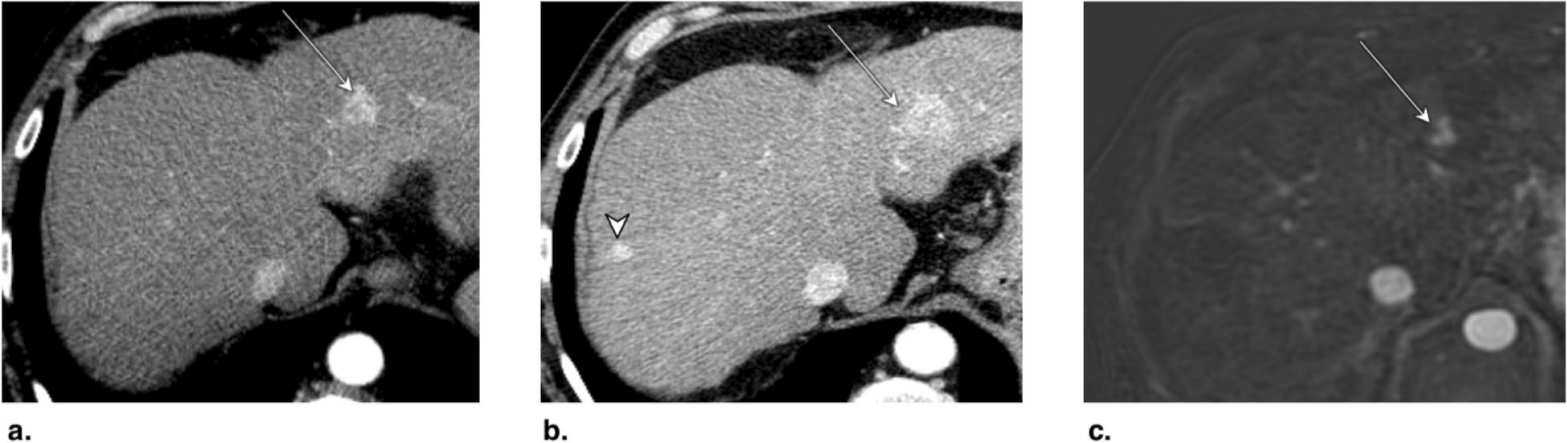
Axial CT in late arterial phase (AP) obtained (**a**) pretreatment: image shows nonrim arterial phase hyperenhancement (APHE) of a 2-cm LR-4 observation in segment II-III (arrow). **b** 2 months post percutaneous ethanol ablation (PEA): image shows interval growth (arrow) and a new LR-4 observation (arrowhead) in segment VIII. At this point, the treated lesion is categorized LR-TR viable. **c** Axial T1-weighted subtraction MR in late AP obtained 1 month post repeat PEA: image shows serpiginous enhancement in the location of the treated lesion possibly representing vascular fistula. The treated observation is categorized LR-TR Equivocal

**Fig. 10:**
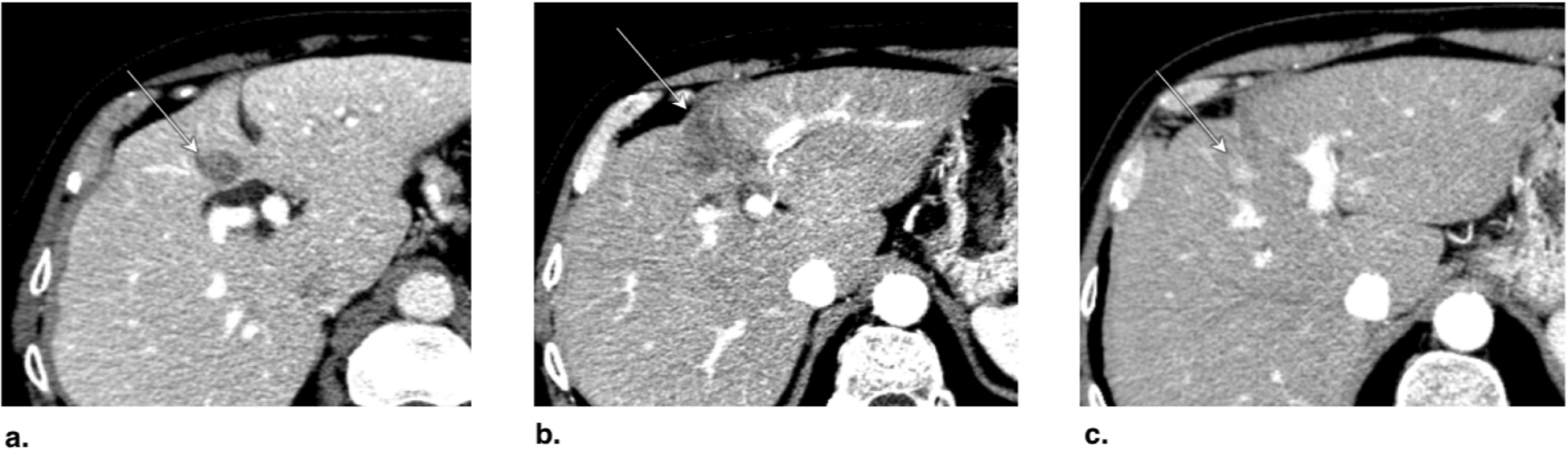
**a** Axial CT in portal venous phase obtained pretreatment: image shows washout of a 2.5-cm LR-5 observation. Proximity to gallbladder and biliary bifurcation contraindicates radiofrequency ablation. Axial CT in late arterial phase obtained 1 month post percutaneous ethanol ablation: images show (**b**) hypoenhancement of the treatment area (arrow), with (**c**) nodular arterial phase hyperenhancement at the upper margin of the treated lesion indicating viable tumor (arrow). The treated observation is categorized LR-TR Viable

**Fig. 11:**
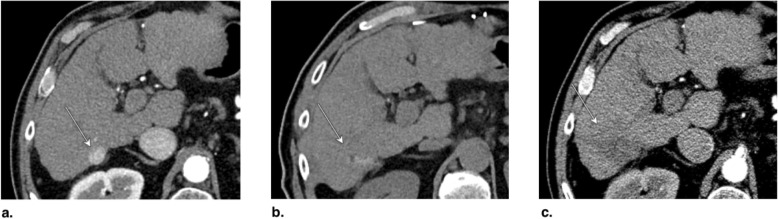
Axial CT obtained (**a**) pretreatment: image in late arterial phase (AP) shows nonrim arterial phase hyperenhancement of a small peripheral LR-5 observation (arrow). **b** Immediately post radiofrequency ablation (RFA): unenhanced image shows hyperdense central cavity related to coagulated blood products (arrow). **c** 3 months post RFA: image in late AP shows unenhanced and enlarged cavity with resorption of blood products. The treated observation is categorized LR-TR Nonviable

**Fig. 12:**
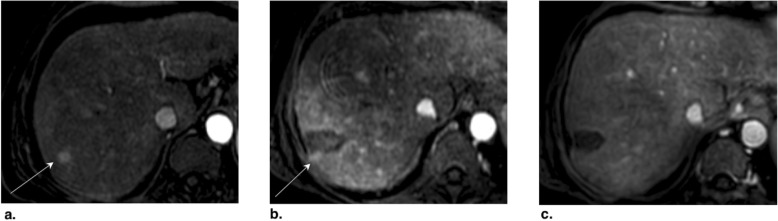
Axial T1-weighted fat-saturated MR in late arterial phase obtained (**a**) pretreatment: image shows nonrim arterial phase hyperenhancement (APHE) of a small HCC (arrow). **b** 1 month post radiofrequency ablation (RFA): image shows irregular rim enhancement and more nodular APHE at posterolateral margin (arrow). The treated observation is categorized LR-TR equivocal. **c** 4 months post RFA: image shows no suspicious enhancement; the treated observation is now categorized LR-TR Nonviable

**Fig. 13:**
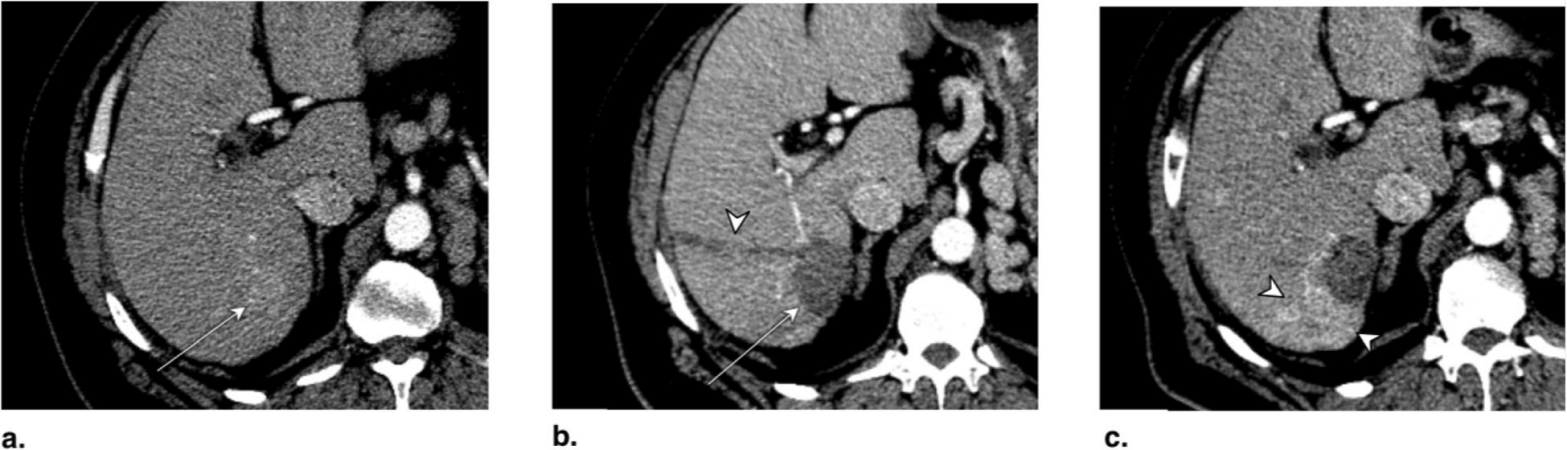
Axial CT in late arterial phase obtained (**a**) pretreatment: image shows nonrim arterial phase hyperenhancement (APHE) of a right lobe LR-4 observation (arrow). **b** 2 months post radiofrequency ablation (RFA): image shows needle trajectory (arrowhead) and a treatment cavity (arrow) with no residual tumor. **c** 8 months post RFA: image shows irregular masslike APHE (arrowheads) posterior to the treatment zone. The treated observation is categorized LR-TR Viable

**Fig. 14:**
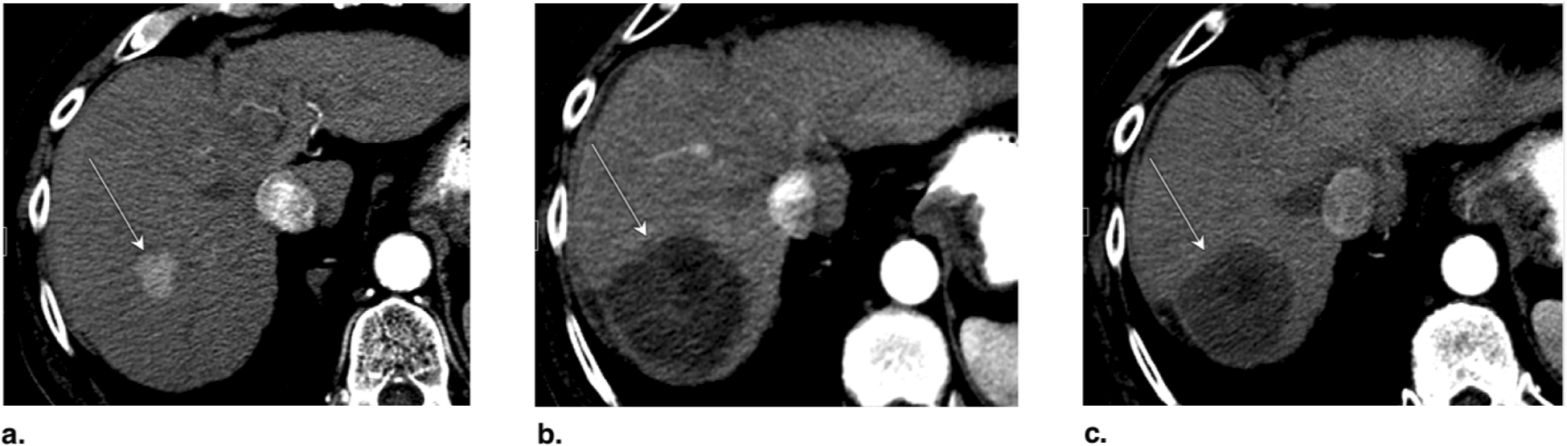
Axial CT in late arterial phase obtained (**a**) pretreatment: image shows nonrim arterial phase hyperenhancement (APHE) of a 2-cm observation in segment VII (arrow). **b** 6 weeks post microwave ablation (MWA): image shows large hypodense cavity (arrow) with margins covering the targeted lesion. **c** 6 months post MWA: image shows well-defined cavity (arrow) with decreased diameter and no APHE. The treated observation is categorized LR-TR Nonviable

**Fig. 15:**
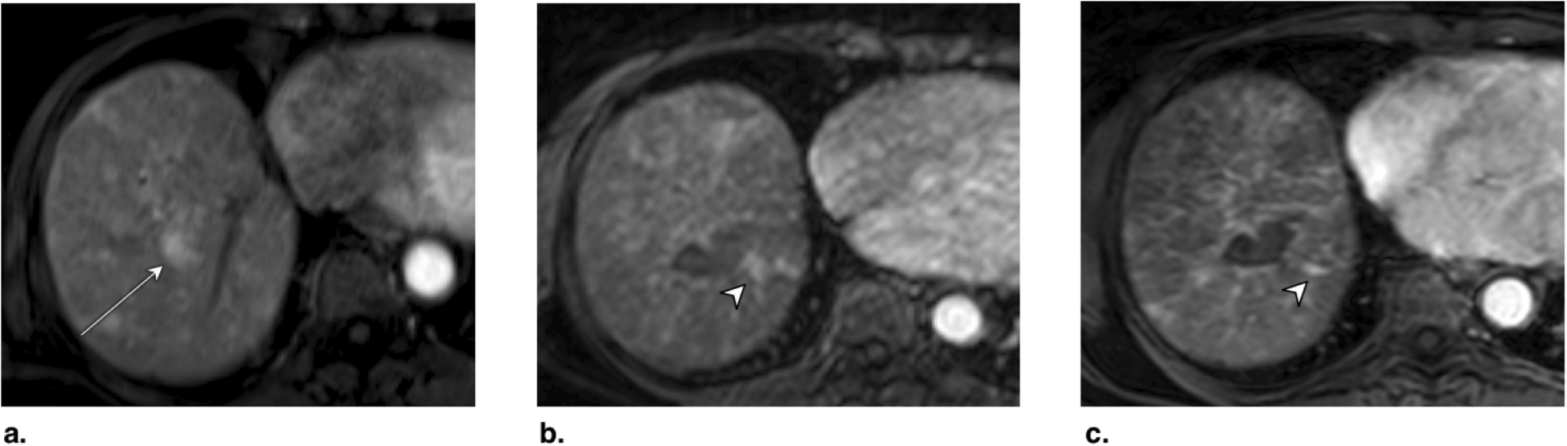
Axial T1-weighted fat-saturated MR in late arterial phase obtained (**a**) pretreatment: image shows arterial phase hyperenhancement (APHE) of a 18-mm LR-5 observation (arrow). **b** 6 weeks post microwave ablation (MWA): image shows two nonspecific APHE perilesional nodules possibly representing perfusional changes (arrowhead). At this point, treated observation is categorized LR-TR equivocal. **c** 3 months post MWA: image shows disappearance of nodules in treatment zone; the treated observation is now categorized LR-TR Nonviable

**Fig. 16:**
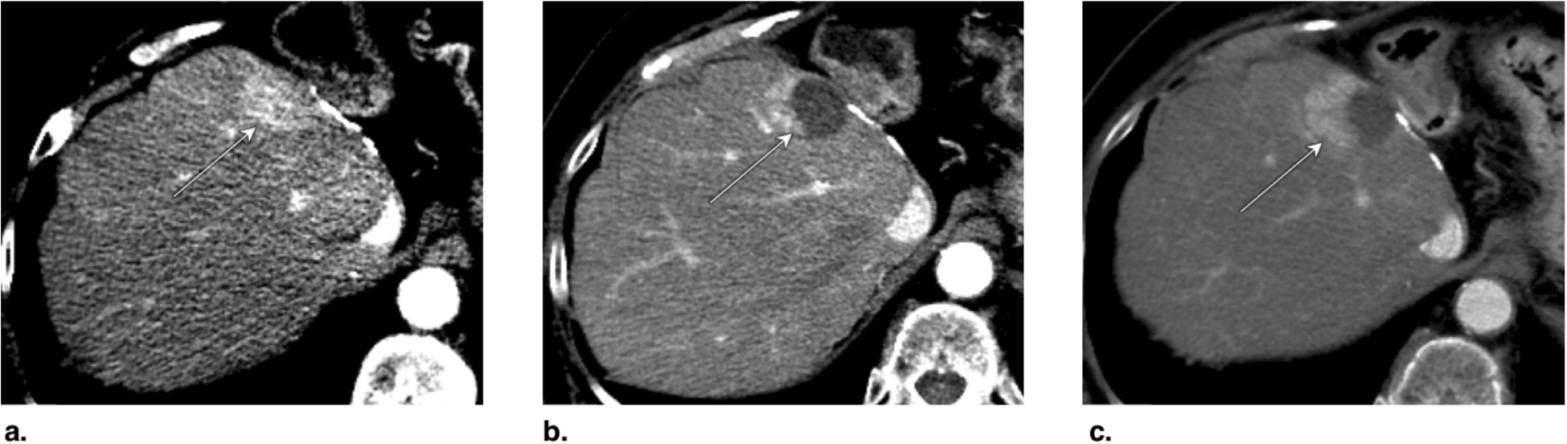
Axial CT in late arterial phase obtained (**a**) pretreatment: image shows nonrim arterial phase hyperenhancement (APHE) of a 2.8-cm LR-5 observation in segment VIII (arrow) of a patient with prior left hepatectomy. **b** 6 weeks post microwave ablation (MWA): image shows hypodense cavity with peripheral thick nodular APHE (arrow). **c** 3 months post MWA: image shows thick nodular APHE (arrow). The treated observation is categorized LR-TR Viable

## Transcatheter therapy

### Technique, mechanism of action, and complications

**Fig. 17:**
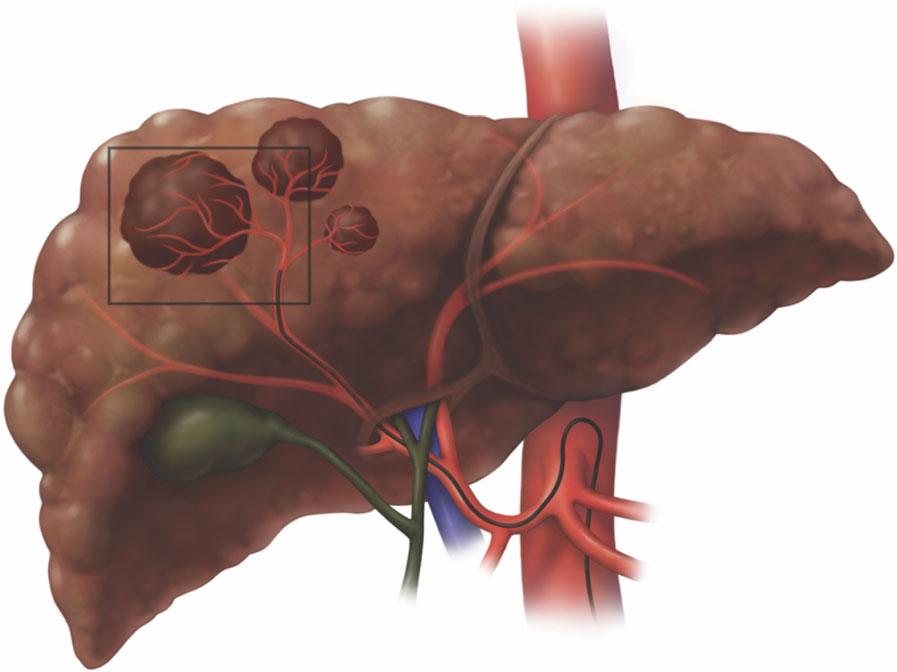
Transcatheter therapy. Tumor arterial blood supply is selectively catheterized to deliver embolic material, chemotherapeutic agent, or radioactive beads

Blood supply of progressed HCC is primarily arterial [37]. Transcatheter delivery of embolic material is intended to selectively obstruct arterial inflow to tumor and induce ischemia and necrosis (Fig. 17). The embolic material may be bland or may contain chemotherapy or radioactive particles. Although arterial supply of a liver segment is compromised after embolization, a patent portal axis compensates hepatocytes perfusion thus reducing damage to healthy liver. Transarterial embolization in the setting of portal thrombosis or invasion is relatively contraindicated although it can be performed if other therapies are unavailable [38].

**Fig. 18:**
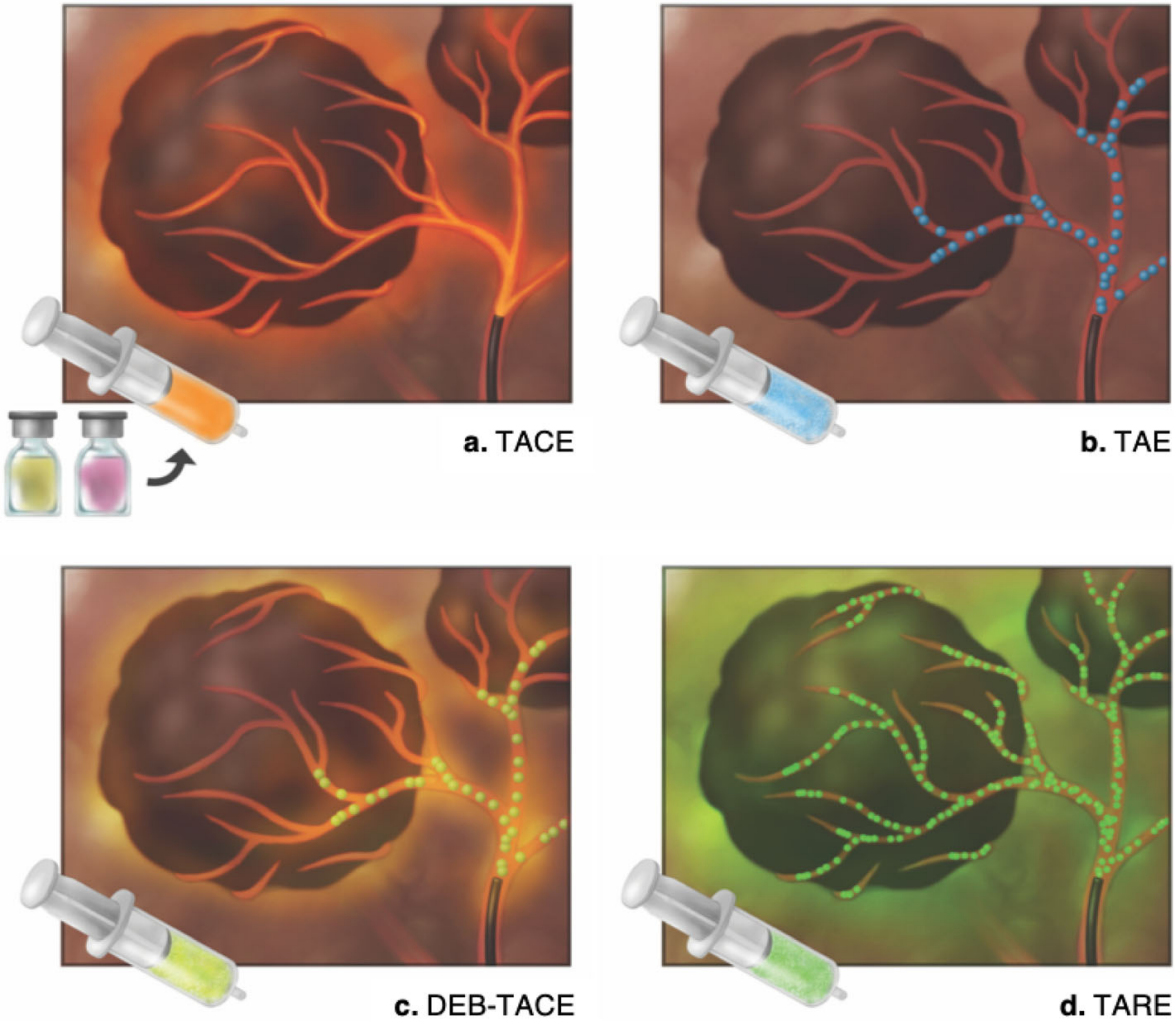
Technically similar, transcatheter therapies greatly differ by the composition of the injection. **a** Transarterial chemoembolization (TACE) uses an emulsion of ethiodized oil and chemotherapy. **b** Transarterial (bland) embolization (TAE) relies solely on bland embolic material to treat the tumor. **c** Drug-eluting beads (DEB-TACE) are loaded with chemotherapeutic agent allowing prolonged delivery. **d** Transarterial radioembolization (TARE) delivers β-emitting microspheres. Note the greater diffusion of administered agents in the tumors with TARE compared to other transcatheter techniques

Four main types of transarterial treatments are used (Fig. 18):

- Transarterial chemoembolization (TACE) is most commonly achieved using a mixture of doxorubicin and ethiodized oil. The oil acts as an emulsifying agent, which enhances embolization efficacy, and is radiopaque, which permits targeting visualization on CT.
- Transarterial (bland) embolization (TAE) relies solely on occlusion of tumor arterial supply with gelatin sponge and microparticles.
- Drug-eluting beads TACE (DEB-TACE) employs hydrogel beads loaded with a chemotherapeutic agent, such as doxorubicin, permitting a higher dose and prolonged delivery of the agent within the tumor [39].
- Transarterial radioembolization (TARE) is carried out using microembolic insoluble glass or resin microspheres loaded with Yttrium-90 (^90^Y). TARE has a minor embolic effect; the main therapeutic action is through radiation. For this reason, TARE is reviewed in the next section (see “Radiation therapy” below).

Regardless of the transcatheter therapy type, aortic, superior mesenteric, and celiac trunk angiography is performed to assess the hepatic vasculature (including anatomical variants) and to identify potential extrahepatic vessel supplying the tumor, arterioportal shunting, and blood flow to other organs [40].

Maintenance of proximal vascular patency is important as multiple treatment sessions may be needed to refine targeting, treat concomitant lesions, or limit tumor growth [41, 42]. When an additional session is necessary, it is typically scheduled at 1 to 6 months. As there is potential for treatment-induced liver failure even without tumor progression, the benefits of additional sessions should be carefully weighted [43, 44].

Transcatheter therapies are well tolerated with 50% of patients discharged the day after treatment [45]. Post-embolization syndrome is the most common side effect (> 40% of patients) observed for as long as 7–10 days posttreatment and includes nausea, fatigue, fever, and abdominal pain [46]. Serious complications (5.6%) are usually due to ischemia of target or non-target organs and include acute liver failure, abscess formation, sepsis, and gastrointestinal bleeding.

Tumor lysis syndrome is a rare complication caused by rapid destruction of neoplastic cells. It occurs most frequently after treatment of large HCC in patients with renal insufficiency and other metabolic derangements (e.g., elevation of uric acid or phosphorous) [47]. Early recognition of this syndrome is important as delay in treatment can be life threatening (e.g., renal insufficiency, cardiac arrythmias, seizure) [47]. Thirty-day mortality is about 1% [48].

### Indications and efficacy

TACE, TAE, and DEB-TACE are non-curative but they may be performed for bridging, debulking, or palliation in patients with unresectable intermediate-stage HCC without vascular invasion [31]. The choice among the various transcatheter therapies is often difficult and should be patient-tailored after multidisciplinary discussion and consensus [31]. Extrinsic factors guiding treatment choice include costs, healthcare policies, and institution’s experience. Broad outline of literature guiding treatment choice is provided below.

TACE has been shown to prolong survival compared to systemic chemotherapy [43, 49]. Although DEB-TACE provides more sustained and tumor-selective drug delivery than conventional TACE [50], a recent meta-analysis did not demonstrate a survival advantage for DEB-TACE over conventional TACE [51]. TAE induces ischemia, which plays a key role in HCC treatment. The benefits of adding a chemotherapeutic agent to a bland embolic agent remains controversial. A meta-analysis concluded that TAE was as effective as TACE [52], and a recent randomized controlled trial did not find a difference in overall survival between DEB-TACE and TAE [45]. Given the high incidence of HCC and costs of chemotherapy, TAE might gain more attention in the future.

### Expected treatment response

After TACE, hyperdensity on unenhanced CT reflects ethiodized oil agent deposition in and around the tumor. Intratumoral ethiodized oil distribution is a CT imaging marker of the distribution of the embolic material within the lesion and thus provides information on how well the lesion was targeted. Despite this benefit, intratumoral oil agent deposition limits assessment of tumor viability on CT because the radiopaque iodized material obscures enhancing viable tumor [30, 53]. MRI helps assess tumor viability because ethiodized oil is not particularly hyperintense on T1-weighted images and so does not mask enhancement. The density and extent of ethiodized oil retention decreases with time. Radiopaque embolic material is not usually used for TAE or DEB-TACE, and the enhancement of viable tumor is not obscured on CT or MRI after these therapies.

TACE, TAE, and DEB-TACE have similar posttreatment appearances except for possible obscuration of enhancement on CT as described above. Early posttreatment, regional hyperenhancement surrounding the tumor may be present, reflecting transient posttreatment perfusional alteration due to inflammation. Thin uniform linear rim enhancement of granulation tissue along the treated zone may persist for months to years. Size of the necrotic zone should decrease over time.

As with locoablative treatment, presence of gas foci within the treated lesion is a frequent finding at first follow-up, being reported in up to 13% of patients at 4–6 weeks follow-up [54], and is a marker of tumor necrosis. Intratumoral gas is rarely associated with abscess. However, patients with a history of bilioenteric anastomosis or sphincterotomy are at higher risks of infection especially if prophylactic antibiotherapy was not administered. A large amount of gas or gas/liquid levels within a cavity larger than the treated tumor are concerning for superinfection [54].

At any point posttreatment, the presence of nodular APHE, washout appearance, or enhancement similar to pretreatment indicates recurrence or residual viable tumor. TARE is discussed in the next section.

The expected treatment response appearances after TACE, TAE, and DEB-TACE are summarized in Figs. 19 and 20. LR-TR nonviable, equivocal, and viable representative CT and MRI cases after TACE (Figs. 21, 22, and 23) and TAE and DEB-TACE (Figs. 24, 25, and 26) are provided.

**Fig. 19:**
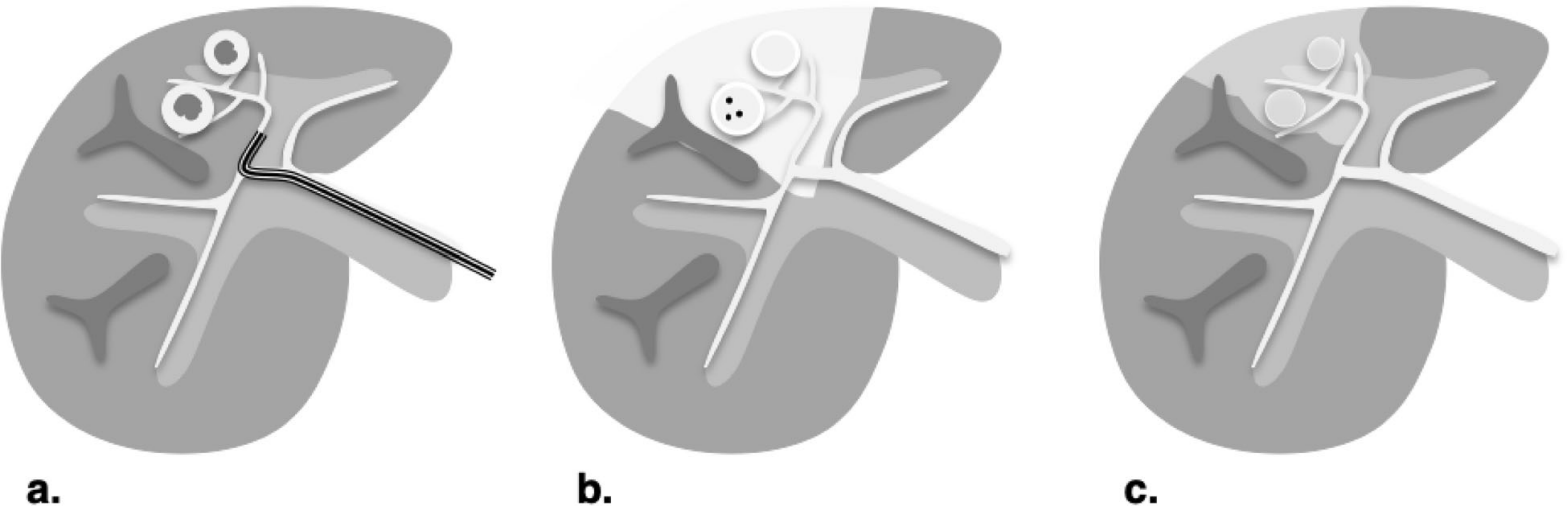
Expected treatment response after transarterial chemoembolization (TACE). Axial contrast-enhanced CT images of the liver obtained in late arterial phase are illustrated: Note: the wedge-shaped hyperdensity on illustrations **b** and **c** reflects the non-target parenchymal ethiodized oil deposition, not enhancement. **a** Pretreatment: typically used for bridging, debulking, or palliative treatment in patients with intermediate-stage hepatocellular carcinoma without vascular invasion. May be used alone or in combination with other treatments. **b** 1–3 months posttreatment: hyperdensity on unenhanced CT reflects ethiodized oil agent deposition in the tumor and at its periphery and reflects distribution of the embolic material. Ethiodized oil limits assessment of viability on CT. MRI helps assessment as oil agent does not mask enhancement. The following features may be seen: thin uniform rim enhancement around the treated zone, regional parenchymal enhancement, and intratumoral gas foci (up to 4–6 weeks posttreatment). **c** ≥ 6 months posttreatment: density and extent of ethiodized oil retention decreases with time. Size of necrotic zone decreases over time. Regional parenchymal enhancement resolves. Rim enhancement around the treated zone may persist for months to years. At any point posttreatment, presence of nodular arterial phase hyperenhancement, washout appearance, or enhancement similar to pretreatment indicates recurrence or residual viable tumor

**Fig. 20:**
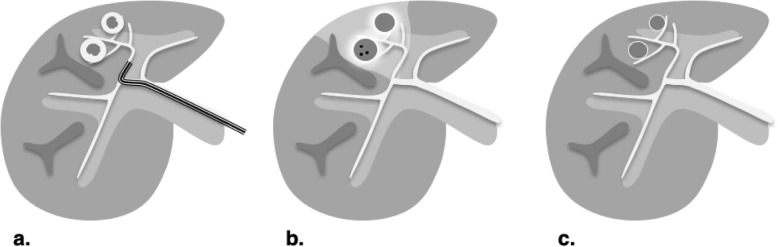
Expected treatment response after transarterial bland embolization (TAE) and drug-eluting beads transarterial chemoembolization (DEB-TACE). Axial contrast-enhanced CT images of the liver obtained in late arterial phase are illustrated: **a** Pretreatment: typically used for bridging, debulking, or palliative treatment in patients with intermediate-stage hepatocellular carcinoma without vascular invasion. May be used alone or in combination with other treatments. **b** 1–3 months posttreatment: TAE and DEB-TACE show similar posttreatment evolution since drug-eluting beads are not visible on imaging. Contrary to TACE, since no hyperdense ethiodized oil is used, tumor viability is easier to assess on CT. The same following features as TACE may be seen: thin uniform rim enhancement around the treated zone, regional parenchymal enhancement, and intratumoral gas foci (up to 4–6 weeks posttreatment). **c** ≥ 6 months posttreatment: size of necrotic zone decreases over time. Regional parenchymal enhancement resolves. Rim enhancement along the treated zone may persist for months to years. At any point posttreatment, presence of nodular arterial phase hyperenhancement, washout appearance, or enhancement similar to pretreatment indicates recurrence or residual viable tumor

**Fig. 21:**
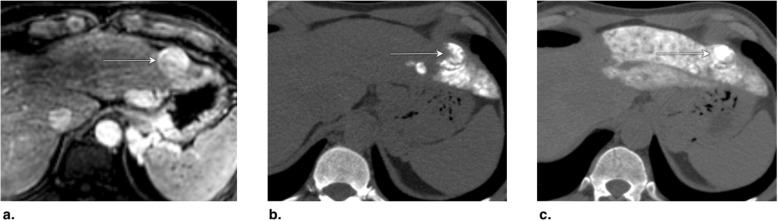
**a** Axial T1-weighted MR in late arterial phase (AP) obtained pretreatment: image shows nonrim arterial phase hyperenhancement (APHE) of a 3-cm LR-5 observation in segments II-III (arrow). **b** Unenhanced axial CT obtained immediately post transarterial chemoembolization (TACE) of segment III: image shows suboptimal targeting with incomplete ethiodized oil retention (arrow). **c** Unenhanced axial CT obtained post repeat TACE: image shows extended treatment area with optimal targeting (arrow). Postcontrast imaging (not shown) did not show APHE or washout, the treated observation is categorized LR-TR Nonviable

**Fig. 22:**
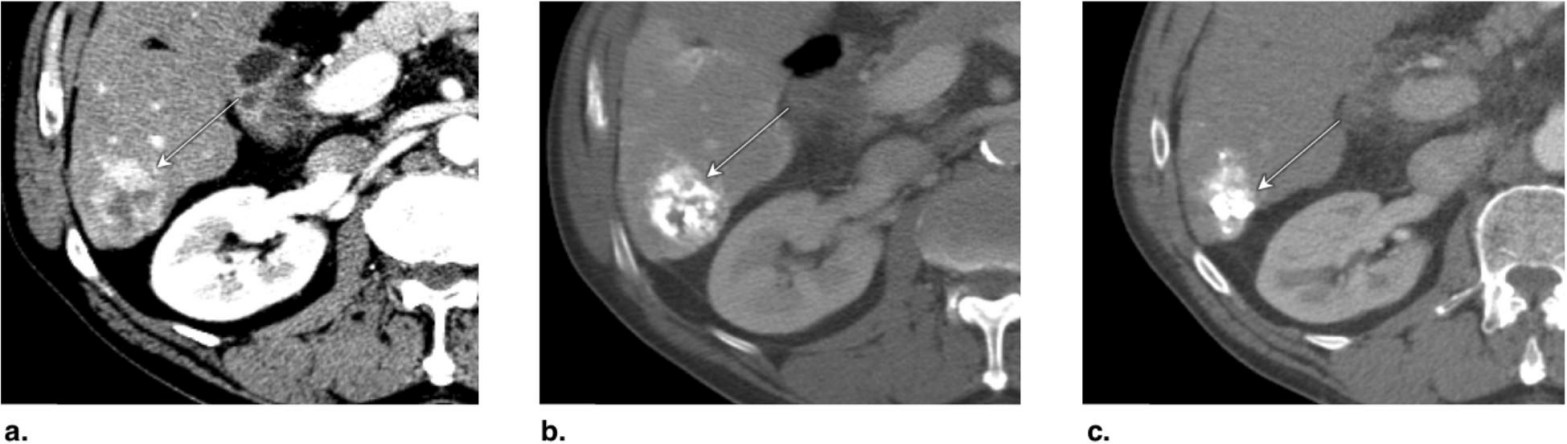
Axial CT obtained (**a**) pretreatment: image in late arterial phase (AP) shows nonrim arterial phase hyperenhancement of a 3.5-cm LR-5 observation in segment VI (arrow). **b** 1 month post transarterial chemoembolization (TACE): image in portal venous phase shows ethiodized oil retention with optimal targeting (arrow). **c** 2 months post TACE: image in late AP shows decrease of ethiodized oil retention (arrow). Density of ethiodized oil limits the assessment at the anterior part of the lesion. The treated observation is categorized LR-TR Equivocal

**Fig. 23:**
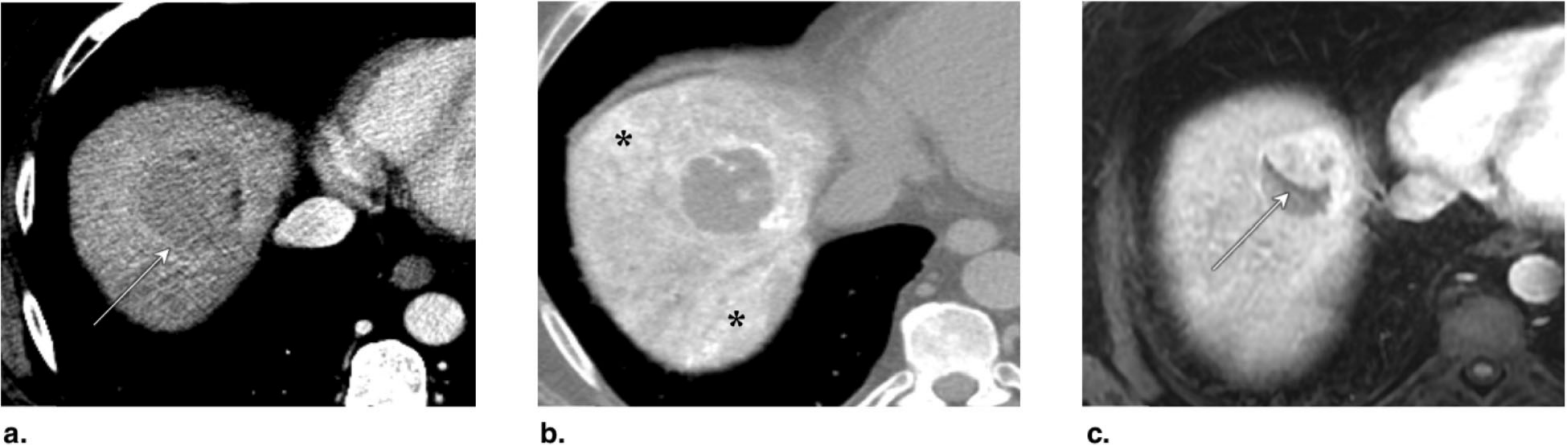
**a** Axial CT in portal venous phase obtained pretreatment: image shows washout of a 4.8-cm LR-5 observation in segment VIII (arrow). **b** Immediately post transarterial chemoembolization (TACE) of right hepatic artery: unenhanced image shows diffuse ethiodized oil retention of the right lobe (asterisks). **c** Axial T1-weighted MR in late AP obtained 4 months post TACE: image shows a 3-cm hyperenhancing nodule at the anterior aspect of the treated lesion. The treated observation is categorized LR-TR Viable

**Fig. 24:**
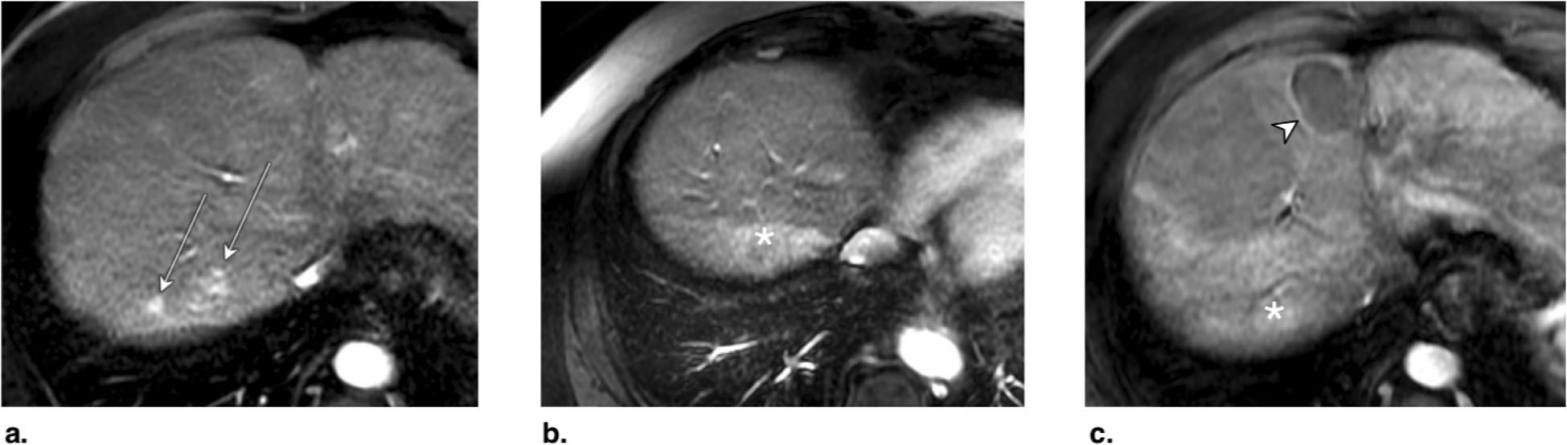
Axial T1-weighted MR in late arterial phase obtained (**a**) pretreatment: image shows two LR-4 observations (arrows) with nonrim arterial phase hyperenhancement (APHE). **b** 2 months post drug-eluting beads TACE (DEB-TACE): image shows regional APHE (asterisk). **c** 10 months post DEB-TACE: image shows decrease in regional APHE (asterisk). No nodular enhancement nor washout is visible. The treated observation is categorized LR-TR nonviable. Note the radiofrequency ablation cavity of another treated lesion (arrowhead)

**Fig. 25:**
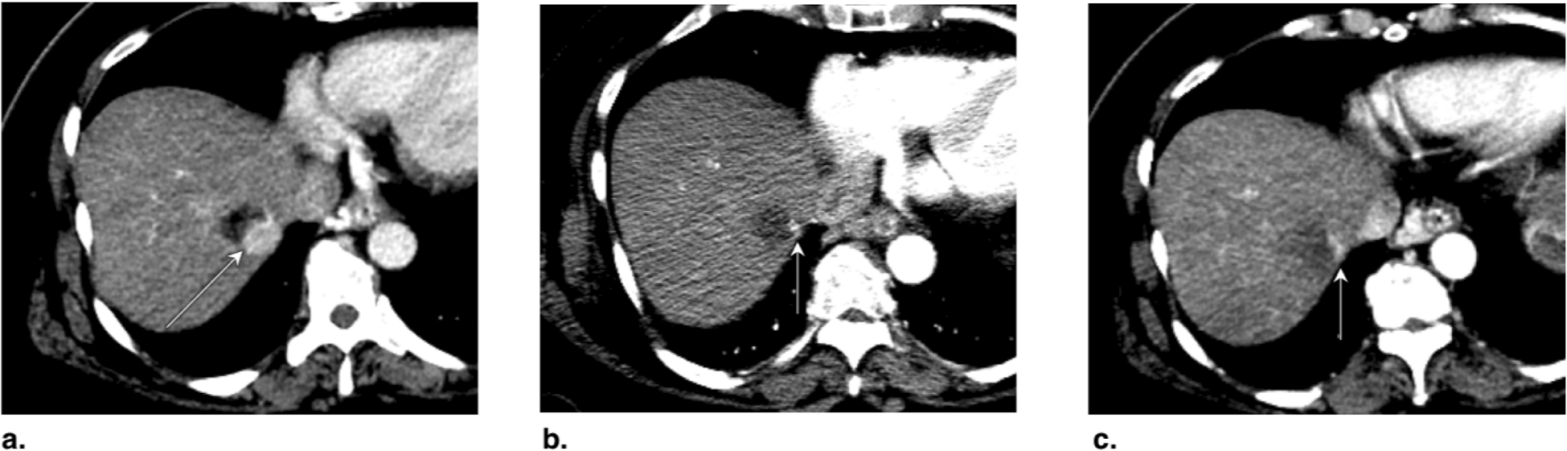
Axial CT in late arterial phase obtained (**a**) pretreatment: image shows arterial phase hyperenhancement (APHE) of a nodule (arrow) within previous drug-eluting beads TACE (DEB-TACE) treatment zone. **b** 1 month post repeat DEB-TACE: image shows APHE of a 3 mm focus (arrow) at periphery of treatment zone. At this point, the treated observation is categorized LR-TR equivocal. **c** 4 months post repeat DEB-TACE: image shows enlarging 14 mm nodular APHE (arrow) at periphery of treatment zone. The treated observation is now categorized LR-TR Viable

**Fig. 26:**
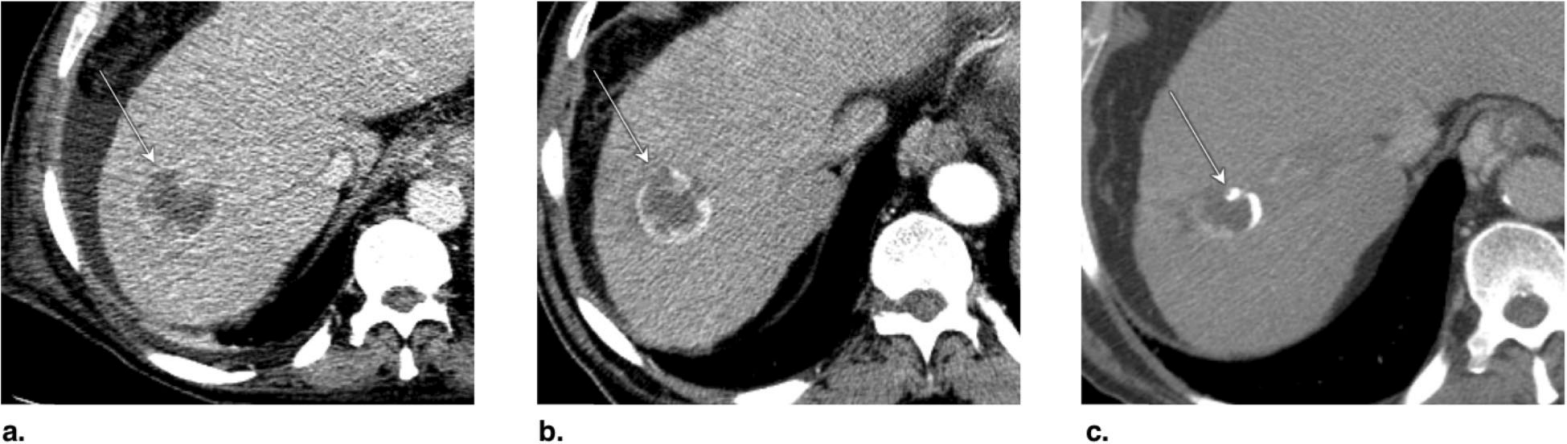
Axial CT obtained (**a**) pretreatment: image in portal venous phase shows enhancing “capsule” and washout of a LR-5 observation in segment VII. **b** 4 months post drug-eluting beads TACE (DEB-TACE): image in late arterial phase shows thicker peripheral arterial phase hyperenhancement (arrow). The treated observation is categorized LR-TR viable. The lesion was subsequently treated with transarterial chemoembolization (TACE). **c** Axial-unenhanced CT post TACE: image shows the expected ethiodized oil retention (arrow)

## Radiation therapy

### Technique, mechanism of action, and complications

HCC is radiosensitive, which explains the underlying rationale for radiation-based therapy. Stereotactic body radiotherapy (SBRT) delivers radiation into the tumor from outside the body whereas TARE delivers internal radiation; however, the underlying cell death mechanism is the same: irreversible damage to the tumor’s vascular endothelium, causing progressive tumor necrosis and tumor size reduction which may continue even after the treatment has ended [55].

#### Stereotactic body radiotherapy

**Fig. 27:**
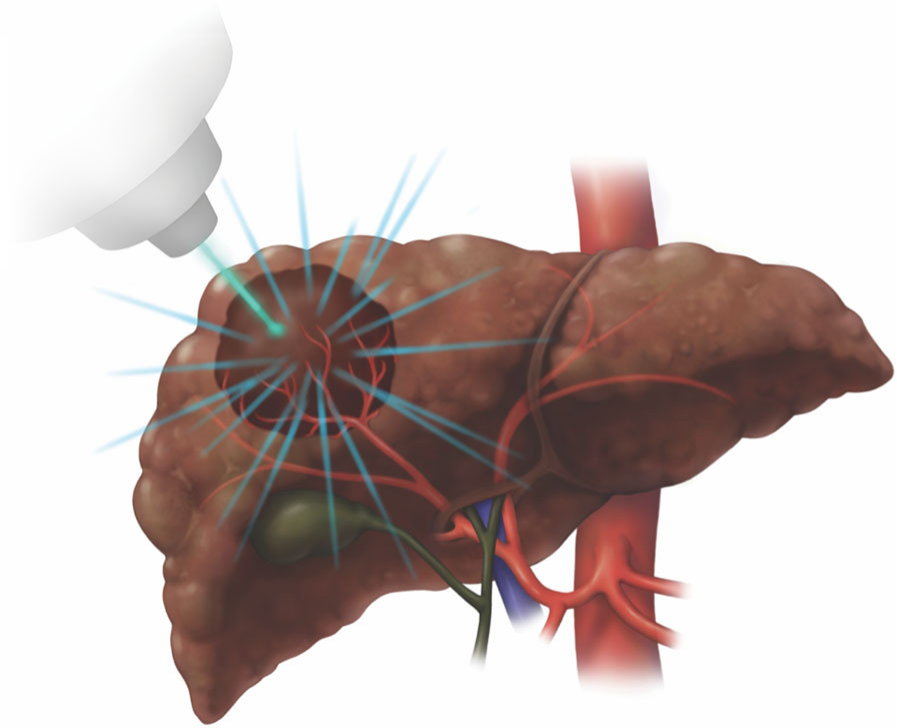
Stereotactic body radiotherapy (SBRT). The tumor is targeted by static conformal beams matching tumor shape and limiting radiation dose to adjacent organs and non-target liver

Stereotactic body radiotherapy (SBRT) delivers focused highly conformal radiation dose distributions (Fig. 27) under image guidance and motion management with rapid dose falloff gradients [34, 56, 57]. This technique enables sparing of large portions of the liver while reaching ablative potential within the tumor [58].

Although most of the liver is spared, some irradiation of non-tumoral liver is unavoidable. Radiation-induced liver disease (RILD), the most significant complication of liver radiotherapy, is a veno-occlusive disease that occurs as an acute- (days to weeks) or a late-response (months to years) after treatment of large hepatic volumes [59]. Manifestations include dysregulated hepatic function, jaundice, and markedly elevated serum transaminases [60]. Other toxicities of SBRT include ulceration, perforation, and stenosis of adjacent hollow viscera (esophagus, stomach, duodenum, intestine), injury to bile ducts, and rarely injury to the spinal cord.

The overall complication rate after SBRT is multifactorial and depends on patients underlying hepatic reserve (greater risk with higher Child-Pugh score) as well as tumor size, number, location, and proximity to critical organs [58, 61, 62]. At 1 to 3 months, 3–44% of patients develop RILD [58, 63] with case fatality rates as high as 5–13% [58, 64].

#### Transarterial radioembolization

All patients scheduled for TARE undergo angiography 1–2 weeks prior to treatment. In addition to the conventional angiography described in the transcatheter therapy section, ^99m^Tc-MAA is injected in the hepatic artery followed by a SPECT or SPECT/CT scan. This procedure aims to determine the expected radiation dose to be delivered to tumor and non-tumor areas and to identify splanchnic and pulmonary shunting [55, 65–67]. Prophylactic embolization of non-target vessels identified with angiography may be performed [68].

Selective delivery of Yttrium-90 (^90^Y) loaded microspheres in the tumor’s vascular supply permits local emission of β-particles (Figs. 17 and 18). These particles have a short 2.4 mm tissue penetration [69]. Despite the heterogenous distribution of microembolic material due to altered liver architecture and hemodynamics, a high radiation dose is preferentially distributed in tumors compared to normal liver parenchyma [70].

Complications of TARE do not result from microembolic effect, but rather from irradiation of non-target tissues including the liver [71]. Incidence of radiation-induced pneumonitis, cholecystitis, and other gastrointestinal complications associated with TARE range from 1 to 5% [66, 67].

After radioembolization, a form of RILD called REILD (radioembolization-induced liver disease), characterized by jaundice and ascites 4–8 weeks after treatment, has been described [72, 73]. This form of veno-occlusive disease has been reported in 8–15% of cirrhotic patients post TARE [72].

### Indications and efficacy

Although further randomized trials are needed, SBRT is increasingly considered a potential alternative to RFA for early-stage HCC [31, 74, 75]. SBRT is also an alternative bridging therapy. A recent retrospective study showed that SBRT provided similar overall survival to TACE and RFA [76]. Dose escalation to achieve better tumor control increases the risk of toxicity, especially in those with cirrhosis and compromised baseline liver function [77]. However, dose-response curves are not well studied for HCC. A systematic review suggests that 90% of local control can be achieved with common fractioned dose regimens [58]. Out-of-field recurrence is an important cause of disease progression [78].

TARE has shown a significant improvement in progression-free survival compared to TACE for early and intermediate-stage HCC [79]. A recent meta-analysis comparing TACE to TARE found an improved survival at 2 and 3 years in the TARE group compared to TACE [80]. Due to its potentially superior antitumoral activity, TARE is currently used in some centers for patients between intermediate and advanced-stage (larger tumors, portal vein invasion, poor candidate for TACE) or poor response post TACE [71]. TARE, among others, has also been used for downstaging patients slightly above resection criteria [81, 82]. Further trials are needed to establish whether TARE can be considered for advanced-stage HCC [31].

### Expected treatment response

Posttreament imaging appearance is similar for radiation-based treatment (SBRT, TARE) but greatly differs from the locoablative and transcatheter therapies using non-radiating embolic material (TACE, TAE, DEB-TACE).

After SBRT, tumor size and enhancement may transiently increase during the first weeks posttreatment. This phenomenon, called pseudoprogression, has not been described with TARE.

After SBRT and TARE, intralesional APHE and washout commonly persists for the first 6 months but should decrease over time. Comparison with prior serial imaging is essential. Geographic and often heterogeneous parenchymal enhancement in the vascular territory of the treated tumor may represent inflammatory hyperemia, venous congestion, and/or radiation fibrosis depending on the timing of imaging after treatment, and can be difficult to differentiate from infiltrative tumor [83].

After 6 months, the treated zone shrinks as radiation fibrosis progresses and APHE and washout usually resolve. Late venous enhancement in the irradiated non-tumorous parenchyma may still be observed after 6 months due to parenchymal fibrosis, along with capsular retraction and biliary dilation. Washout may help differentiate radiation-induced changes from tumor progression [59].

Intralesional enhancement and washout appearance may persist for several months but eventually regress. An increase in enhancement or in washout appearance after an initial favorable response suggests recurrence.

**Fig. 28:**
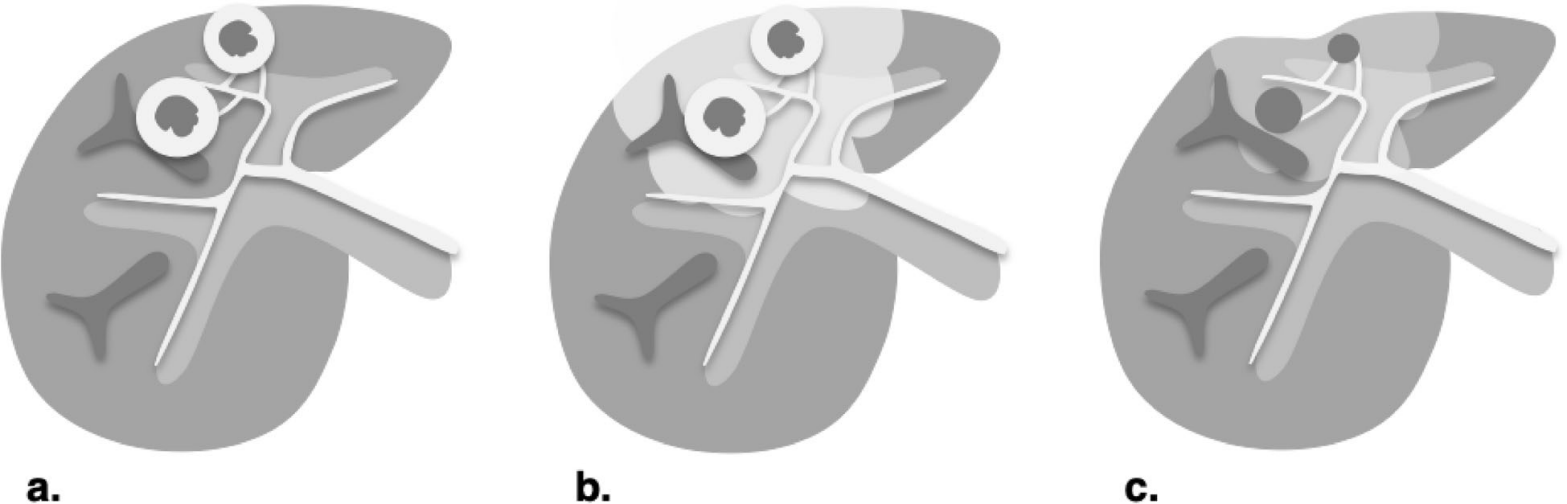
Expected treatment response after stereotactic body radiotherapy (SBRT) and transarterial radioembolization (TARE). Axial contrast-enhanced CT of the liver obtained in late arterial phase are illustrated: **a** Pretreatment: typically used for bridging, debulking, or palliative treatment in patients with intermediate- to advanced-stage hepatocellular carcinoma. May be used alone or in combination with other treatments. SBRT may be used as an alternative to RFA for early-stage HCC. With SBRT, lesions should be located away from critical organs. Before TARE, ^99m^Tc-MAA scan is performed to determine radiation dose to be delivered to tumor/non-tumor areas and identify shunting. **b** 1–3 months posttreatment: intralesional nodular arterial phase hyperenhancement and washout may persist but should gradually fade as radiation necrosis progresses. Geographic enhancing region surrounding the treated zone may represent inflammatory hyperemia, venous congestion, and radiation fibrosis and could be misinterpreted as infiltrative disease. With SBRT, tumor size and enhancement may transiently increase during the first weeks posttreatment, a phenomenon called pseudoprogression. **c** ≥ 6 months posttreatment: treated zone shrinks as fibrosis progresses and is associated with capsular retraction. Intralesional enhancement and washout appearance may persist but usually resolves after 6 months. Late venous enhancement in the irradiated non-tumorous parenchyma may still be observed. Washout may help differentiate radiation-induced changes from tumor progression. An increase in enhancement or in washout appearance after an initial favorable response suggests recurrence

**Fig. 29:**
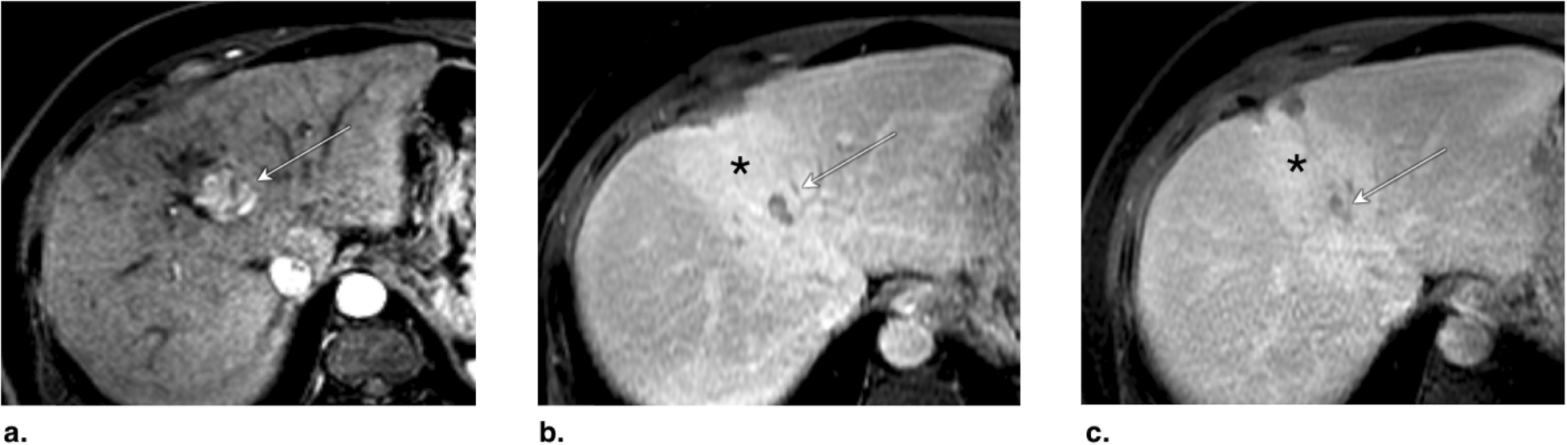
Axial T1-weighted MR obtained (**a**) pretreatment: image in late arterial phase shows hyperenhancement of a 2.5 cm LR-5 observation. **b** 6 months post stereotactic body radiotherapy (SBRT): image in portal venous phase shows persistent 7-mm nodular enhancement (arrow), perfusional anomalies (asterisk), and capsular retraction. **c** 1 year post SBRT: image in portal venous phase shows absence of nodular enhancement and persistent but decreased perfusional anomalies. The treated observation is categorized LR-TR Nonviable

**Fig. 30:**
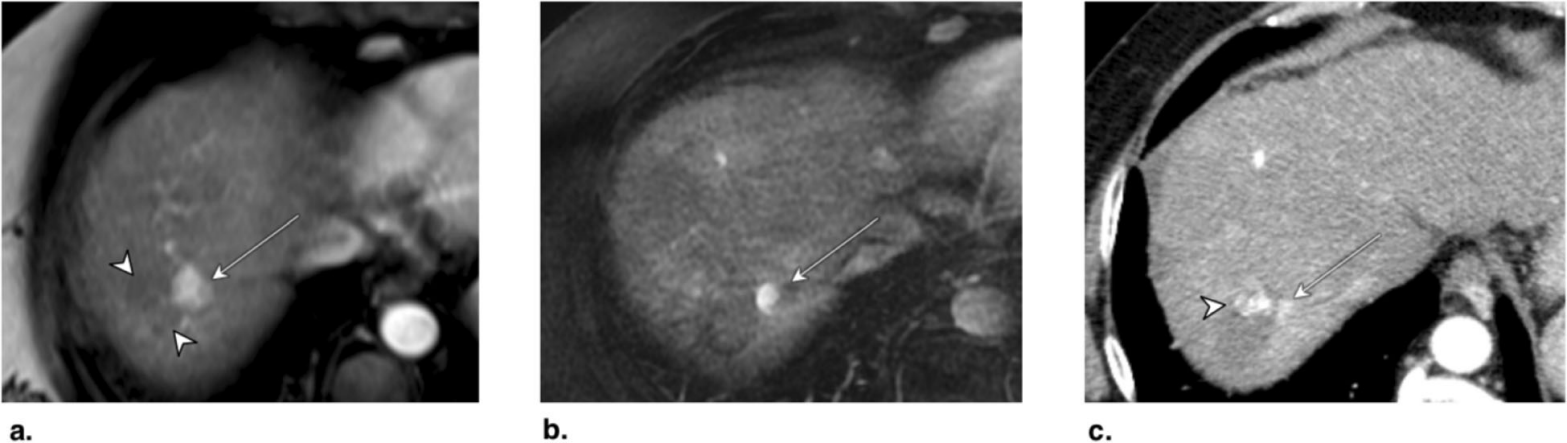
Axial T1-weighted MR in late arterial phase (AP) obtained (**a**) pretreatment: image shows recurrence (arrow) of a lesion previously treated with radiofrequency and transarterial chemoembolization (TACE) (arrowheads). **b** 2 months post stereotactic body radiotherapy (SBRT): image shows incomplete tumor regression. **c** Axial CT in late AP obtained 5 months post SBRT: image shows faint residual enhancement (arrow) that may represent expected enhancement pattern. The treated observation is categorized LR-TR equivocal. Note the ethiodized oil retention from previous TACE (arrowhead)

**Fig. 31:**
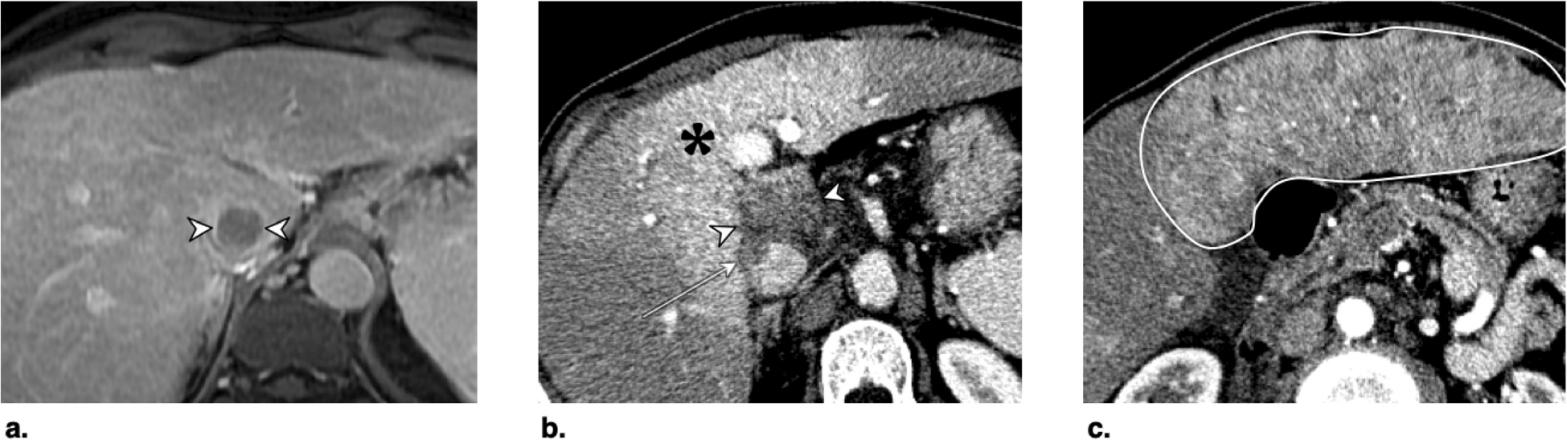
**a** Axial T1-weighted MR in portal venous phase obtained pretreatment: image shows washout of a 2.5 cm LR-5 observation in caudate lobe (arrow). **b** Axial CT in late arterial phase (AP) obtained 3 months post stereotactic body radiotherapy (SBRT): image shows tumor progression (arrowheads) and perfusional anomalies related to edema (asterisk). Mural thrombus in inferior vena cava is noted (arrow). The treated observation is categorized LR-TR viable. **c** Axial CT in late AP obtained 6 months posttreatment: image shows diffuse tumoral infiltration of left lobe (arrow)

**Fig. 32:**
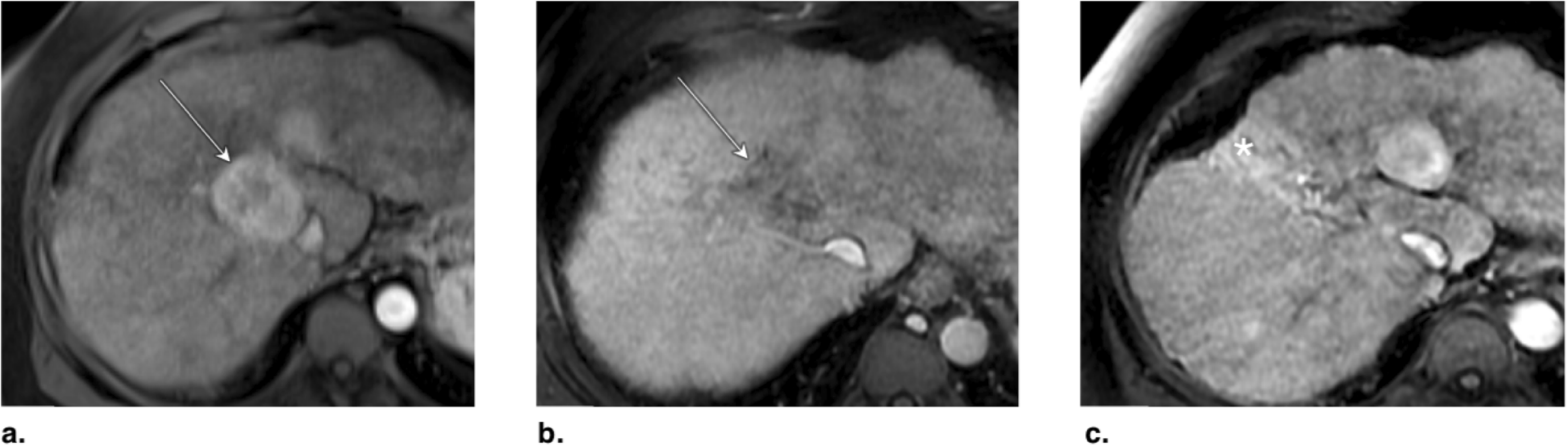
Axial T1-weighted MR obtained (**a**) pretreatment: image in late arterial phase (AP) shows a 5-cm LR-5 observation (arrow). **b** 1 month post transarterial radioembolization (TARE): image in portal venous phase shows hypoenhancing area. **c** 1 year post TARE: image in late AP shows expected capsular retraction and perfusional anomalies (asterisk). The treated observation is categorized LR-TR Nonviable

**Fig. 33:**
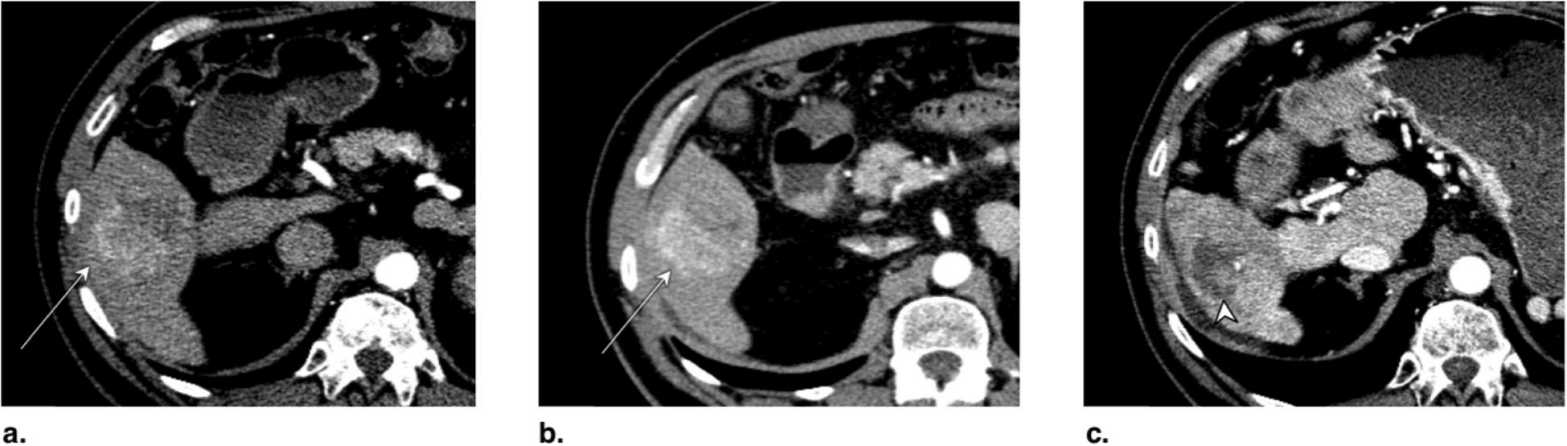
Axial CT in late arterial phase obtained (**a**) pretreatment: image shows an enhancing 6-cm LR-5 observation in segment VI (arrows). **b** 1 month post transarterial radioembolization (TARE): image shows no change. **c** 6 months post TARE: image shows atrophy and diffuse enhancement of segment VI, hypoenhancement at the anterior aspect of the lesion and faint posterior enhancement (arrowhead). These changes may represent treatment-specific expected enhancement pattern or residual tumor. The treated observation is categorized LR-TR Equivocal

**Fig. 34:**
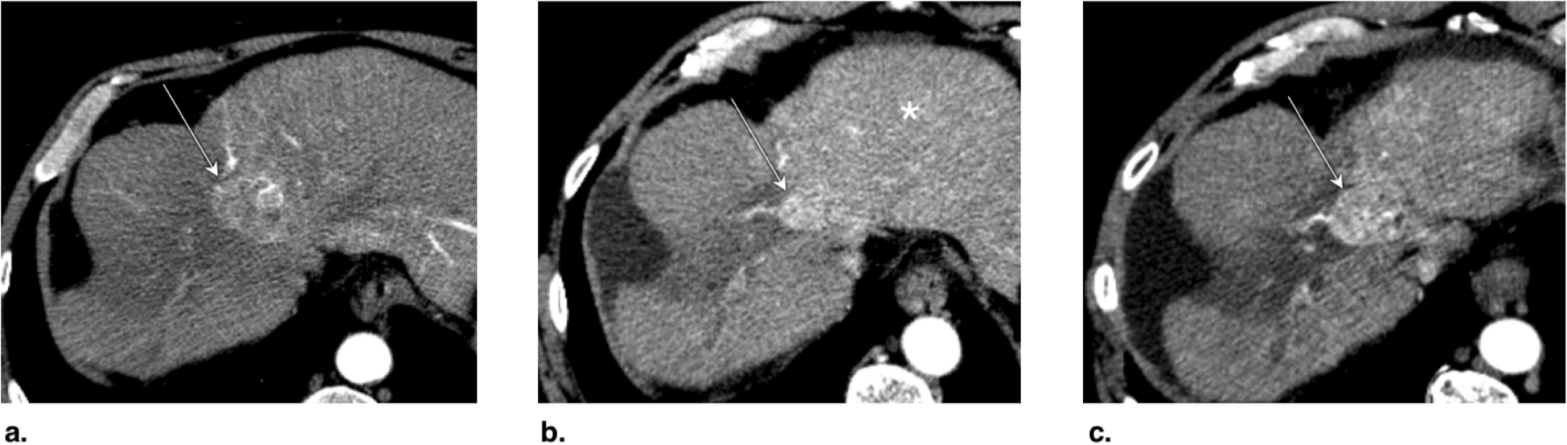
Axial CT in late arterial phase obtained (**a**) pretreatment: image shows a 4-cm LR-5 observation (arrow) in segment IVa. **b** 3 months post left lobar transarterial radioembolization (TARE): image shows tumor regression and perfusional changes in left liver (asterisk) related to TARE. **c** 6 months post TARE: image shows tumor progression (arrow) compatible with recurrence. The treated observation is categorized LR-TR Viable

The expected treatment response appearances after SBRT and TARE are summarized in Fig. 28. LR-TR nonviable, equivocal, and viable representative CT and MRI cases after SBRT (Figs. 29, 30, and 31) and TARE (Figs. 32, 33, and 34) are provided.

## Management recommendations

LI-RADS provides the following management suggestions by AASLD and LI-RADS in consensus for treated observation categories:

- LR-TR Nonevaluable, LR-TR Nonviable, or LR-TR Equivocal: continue monitoring in ≤ 3 months with the same modality or different modality.
- LR-TR Viable: multidisciplinary discussion for consensus management. Often includes retreatment.

## Pitfalls on posttreatment imaging

The current LR-TR algorithm imaging criteria rely mainly on enhancement pattern (APHE, washout appearance, or enhancement similar to pretreatment) to assign a response category. In posttreatment setting, ancillary features can help detection, increase reader confidence, or help characterize untreated observations. A detailed discussion of ancillary features for diagnosis of HCC is beyond the scope of this manuscript [84, 85].

At MRI, T1-weighted hyperintensity representing coagulative necrosis can be present after several types of locoregional treatments and may mimic or mask enhancement. Therefore, subtraction images may be helpful to differentiate true enhancement from pseudo-enhancement due to intrinsic T1 hyperintensity of coagulation necrosis.

Ethiodized oil retention may mask enhancement at follow-up CT after TACE. Unenhanced CT may help differentiating tumor from ethiodized oil uptake. For challenging cases, MRI should be considered as enhancement is not confounded by ethiodized oil.

Perilesional APHE is suspicious for viable tumor depending on its morphology, but is more challenging in the early posttreatment period with SBRT and TARE. When in doubt, the LR-TR Equivocal category should be applied.

Multimodality treatment is frequently performed, each affecting the liver parenchyma in its own manner. A thorough review of patient’s treatment history and awareness of pretreatment appearance is required for appropriate interpretation.

If unsure between categories (LR-TR Nonviable, Equivocal, Viable), LR-TR algorithm recommends choosing LR-TR Equivocal to reflect the uncertainty in posttreatment imaging.

## Conclusions

A number of locoregional HCC treatments are available and their use varies according to the tumor burden, tumor location, patient’s comorbidities, and institutional preference and expertise. Assessment of treatment response needs to consider expected imaging findings that may vary with the type of treatment, the magnitude of treatment response, and the timing of imaging after treatment. Having a basic knowledge of the existing treatment modalities and their expected appearance facilitates assessment of treatment response. LI-RADS Treatment Response is a practical way to assess and report treatment response. The four LR-TR categories are: LR-TR Nonevaluable, Nonviable, Equivocal, and Viable.

## Data Availability

Data sharing is not applicable to this article as no datasets were generated or analyzed during the current study.

## Abbreviations

AP: Arterial phase
APHE: Arterial phase hyperenhancement
CT: Computed tomography
DEB-TACE: Drug-eluting beads TACE
HCC: Hepatocellular carcinoma
LI-RADS: Liver Imaging Reporting and Data System
LR-TR: LI-RADS Treatment Response
MRI: Magnetic resonance imaging
MWA: Microwave ablation
PEA: Percutaneous ethanol ablation
RFA: Radiofrequency ablation
RILD: Radiation-induced liver disease
SBRT: Stereotactic body radiation therapy
TACE: Transarterial chemoembolization
TAE: Transarterial bland embolization
TARE: Transarterial radioembolization

## References

[1] Villanueva A (2019). Hepatocellular carcinoma. N Engl J Med.

[2] Kielar Ania, Fowler Kathryn J., Lewis Sara, Yaghmai Vahid, Miller Frank H., Yarmohammadi Hooman, Kim Charles, Chernyak Victoria, Yokoo Takeshi, Meyer Jeffrey, Newton Isabel, Do Richard K. (2017). Locoregional therapies for hepatocellular carcinoma and the new LI-RADS treatment response algorithm. Abdominal Radiology.

[3] Tang An, Fowler Kathryn J., Chernyak Victoria, Chapman William C., Sirlin Claude B. (2017). LI-RADS and transplantation for hepatocellular carcinoma. Abdominal Radiology.

[4] Chernyak V, Fowler KJ, Kamaya A (2018). Liver Imaging Reporting and Data System (LI-RADS) version 2018: imaging of hepatocellular carcinoma in at-risk patients. Radiology.

[5] American College of Radiology (2018) Liver Imaging Reporting and Data System version 2018 Manual. Available via: https://www.acr.org/-/media/ACR/Files/Clinical-Resources/LIRADS/LI-RADS-2018-Manual-5Dec18.pdf?la=en. Accessed 11th June 2019.

[6] Ahmed M, Solbiati L, Brace CL (2014). Image-guided tumor ablation: standardization of terminology and reporting criteria--a 10-year update. Radiology.

[7] Gaba RC, Lewandowski RJ, Hickey R (2016). Transcatheter therapy for hepatic malignancy: standardization of terminology and reporting criteria. J Vasc Interv Radiol.

[8] Guan YS, Sun L, Zhou XP, Li X, Zheng XH (2004). Hepatocellular carcinoma treated with interventional procedures: CT and MRI follow-up. World J Gastroenterol.

[9] Joseph F B, Baumgarten D A, Bernardino M E (1993). Hepatocellular carcinoma: CT appearance after percutaneous ethanol ablation therapy. Work in progress. Radiology.

[10] Lin SM, Lin CJ, Lin CC, Hsu CW, Chen YC (2005). Randomised controlled trial comparing percutaneous radiofrequency thermal ablation, percutaneous ethanol injection, and percutaneous acetic acid injection to treat hepatocellular carcinoma of 3 cm or less. Gut.

[11] Shiina S, Teratani T, Obi S (2005). A randomized controlled trial of radiofrequency ablation with ethanol injection for small hepatocellular carcinoma. Gastroenterology.

[12] Stauffer P. R., Goldberg S. N. (2004). Introduction: Thermal ablation therapy. International Journal of Hyperthermia.

[13] Goldberg SN, Gazelle GS, Mueller PR (2000). Thermal ablation therapy for focal malignancy: a unified approach to underlying principles, techniques, and diagnostic imaging guidance. AJR Am J Roentgenol.

[14] Thompson SM, Callstrom MR, Butters KA (2014). Heat stress induced cell death mechanisms in hepatocytes and hepatocellular carcinoma: in vitro and in vivo study. Lasers Surg Med.

[15] Goldberg SN, Stein MC, Gazelle GS, Sheiman RG, Kruskal JB, Clouse ME (1999). Percutaneous radiofrequency tissue ablation: optimization of pulsed-radiofrequency technique to increase coagulation necrosis. J Vasc Interv Radiol.

[16] Lin ZY, Li GL, Chen J, Chen ZW, Chen YP, Lin SZ (2016). Effect of heat sink on the recurrence of small malignant hepatic tumors after radiofrequency ablation. J Cancer Res Ther.

[17] Poulou LS, Botsa E, Thanou I, Ziakas PD, Thanos L (2015). Percutaneous microwave ablation vs radiofrequency ablation in the treatment of hepatocellular carcinoma. World J Hepatol.

[18] Simon CJ, Dupuy DE, Mayo-Smith WW (2005). Microwave ablation: principles and applications. Radiographics.

[19] Wright AS, Lee FT, Mahvi DM (2003). Hepatic microwave ablation with multiple antennae results in synergistically larger zones of coagulation necrosis. Ann Surg Oncol.

[20] Mulier S, Mulier P, Ni Y (2002). Complications of radiofrequency coagulation of liver tumours. Br J Surg.

[21] Nakagomi R, Tateishi R, Shiina S (2014). Drastically reduced neoplastic seeding related to radiofrequency ablation for hepatocellular carcinoma. Am J Gastroenterol.

[22] Rhim H (2005). Complications of radiofrequency ablation in hepatocellular carcinoma. Abdom Imaging.

[23] Yang B, Zan RY, Wang SY (2015). Radiofrequency ablation versus percutaneous ethanol injection for hepatocellular carcinoma: a meta-analysis of randomized controlled trials. World J Surg Oncol.

[24] Brunello F, Veltri A, Carucci P (2008). Radiofrequency ablation versus ethanol injection for early hepatocellular carcinoma: a randomized controlled trial. Scand J Gastroenterol.

[25] Lin SM, Lin CJ, Lin CC, Hsu CW, Chen YC (2004). Radiofrequency ablation improves prognosis compared with ethanol injection for hepatocellular carcinoma < or =4 cm. Gastroenterology.

[26] Lu MD, Kuang M, Liang LJ (2006). Surgical resection versus percutaneous thermal ablation for early-stage hepatocellular carcinoma: a randomized clinical trial. Zhonghua Yi Xue Za Zhi.

[27] Chen MS, Li JQ, Zheng Y (2006). A prospective randomized trial comparing percutaneous local ablative therapy and partial hepatectomy for small hepatocellular carcinoma. Ann Surg.

[28] Kutlu OC, Chan JA, Aloia TA (2017). Comparative effectiveness of first-line radiofrequency ablation versus surgical resection and transplantation for patients with early hepatocellular carcinoma. Cancer.

[29] Xu XL, Liu XD, Liang M, Luo BM (2017) Radiofrequency ablation versus hepatic resection for small hepatocellular carcinoma: systematic review of randomized controlled trials with meta-analysis and trial sequential analysis. Radiology. 10.1148/radiol.2017162756:162756 10.1148/radiol.2017162756 29135366

[30] European Association for the Study of the Liver (2018) EASL clinical practice guidelines: management of hepatocellular carcinoma. J Hepatol 69:182–236 10.1016/j.jhep.2018.03.019 29628281

[31] Marrero JA, Kulik LM, Sirlin CB (2018). Diagnosis, staging, and management of hepatocellular carcinoma: 2018 practice guidance by the American Association for the Study of Liver Diseases. Hepatology.

[32] Vietti Violi N, Duran R, Guiu B (2018). Efficacy of microwave ablation versus radiofrequency ablation for the treatment of hepatocellular carcinoma in patients with chronic liver disease: a randomised controlled phase 2 trial. Lancet Gastroenterol Hepatol.

[33] Lencioni R, de Baere T, Martin RC, Nutting CW, Narayanan G (2015). Image-guided ablation of malignant liver tumors: recommendations for clinical validation of novel thermal and non-thermal technologies - a Western perspective. Liver Cancer.

[34] Kielar Ania, Fowler Kathryn J., Lewis Sara, Yaghmai Vahid, Miller Frank H., Yarmohammadi Hooman, Kim Charles, Chernyak Victoria, Yokoo Takeshi, Meyer Jeffrey, Newton Isabel, Do Richard K. (2017). Locoregional therapies for hepatocellular carcinoma and the new LI-RADS treatment response algorithm. Abdominal Radiology.

[35] Oei T, vanSonnenberg E, Shankar S, Morrison PR, Tuncali K, Silverman SG (2005). Radiofrequency ablation of liver tumors: a new cause of benign portal venous gas. Radiology.

[36] Kim KW, Lee JM, Choi BI (2011). Assessment of the treatment response of HCC. Abdom Imaging.

[37] Park YN, Yang CP, Fernandez GJ, Cubukcu O, Thung SN, Theise ND (1998). Neoangiogenesis and sinusoidal “capillarization” in dysplastic nodules of the liver. Am J Surg Pathol.

[38] Yasui D, Murata S, Ueda T (2018). Novel treatment strategy for advanced hepatocellular carcinoma: combination of conventional transcatheter arterial chemoembolization and modified method with portal vein occlusion for cases with arterioportal shunt: a preliminary study. Acta Radiol.

[39] Lammer J, Malagari K, Vogl T (2010). Prospective randomized study of doxorubicin-eluting-bead embolization in the treatment of hepatocellular carcinoma: results of the PRECISION V study. Cardiovasc Intervent Radiol.

[40] Liu DM, Salem R, Bui JT (2005). Angiographic considerations in patients undergoing liver-directed therapy. J Vasc Interv Radiol.

[41] Lee EW, Khan S (2017). Recent advances in transarterial embolotherapies in the treatment of hepatocellular carcinoma. Clin Mol Hepatol.

[42] Terzi E, Ray Kim W, Sanchez W (2015). Impact of multiple transarterial chemoembolization treatments on hepatocellular carcinoma for patients awaiting liver transplantation. Liver Transpl.

[43] Llovet JM, Bruix J (2003). Systematic review of randomized trials for unresectable hepatocellular carcinoma: chemoembolization improves survival. Hepatology.

[44] Raoul JL, Gilabert M, Piana G (2014). How to define transarterial chemoembolization failure or refractoriness: a European perspective. Liver Cancer.

[45] Brown KT, Do RK, Gonen M (2016). Randomized trial of hepatic artery embolization for hepatocellular carcinoma using doxorubicin-eluting microspheres compared with embolization with microspheres alone. J Clin Oncol.

[46] Sangro B (2014). Chemoembolization and radioembolization. Best Pract Res Clin Gastroenterol.

[47] Zivin SP, Elias Y, Ray CE (2015). Tumor lysis syndrome and primary hepatic malignancy: case presentation and review of the literature. Semin Intervent Radiol.

[48] Brown DB, Geschwind JF, Soulen MC, Millward SF, Sacks D (2009). Society of Interventional Radiology position statement on chemoembolization of hepatic malignancies. J Vasc Interv Radiol.

[49] Lo CM, Ngan H, Tso WK (2002). Randomized controlled trial of transarterial lipiodol chemoembolization for unresectable hepatocellular carcinoma. Hepatology.

[50] Lewis AL, Gonzalez MV, Lloyd AW (2006). DC bead: in vitro characterization of a drug-delivery device for transarterial chemoembolization. J Vasc Interv Radiol.

[51] Facciorusso A, Mariani L, Sposito C (2016). Drug-eluting beads versus conventional chemoembolization for the treatment of unresectable hepatocellular carcinoma. J Gastroenterol Hepatol.

[52] Marelli L, Stigliano R, Triantos C (2007). Transarterial therapy for hepatocellular carcinoma: which technique is more effective? A systematic review of cohort and randomized studies. Cardiovasc Intervent Radiol.

[53] Kwan SW, Fidelman N, Ma E, Kerlan RK, Yao FY (2012). Imaging predictors of the response to transarterial chemoembolization in patients with hepatocellular carcinoma: a radiological-pathological correlation. Liver Transpl.

[54] Bisseret D, Ronot M, Abdel-Rehim M (2013). Intratumoral gas in hepatocellular carcinoma following transarterial chemoembolization: associated factors and clinical impact. J Vasc Interv Radiol.

[55] Salem R, Mazzaferro V, Sangro B (2013). Yttrium 90 radioembolization for the treatment of hepatocellular carcinoma: biological lessons, current challenges, and clinical perspectives. Hepatology.

[56] Park MJ, Kim SY, Yoon SM (2014). Stereotactic body radiotherapy-induced arterial hypervascularity of non-tumorous hepatic parenchyma in patients with hepatocellular carcinoma: potential pitfalls in tumor response evaluation on multiphase computed tomography. PLoS One.

[57] Potters L, Kavanagh B, Galvin JM (2010). American Society for Therapeutic Radiology and Oncology (ASTRO) and American College of Radiology (ACR) practice guideline for the performance of stereotactic body radiation therapy. Int J Radiat Oncol Biol Phys.

[58] Schaub SK, Hartvigson PE, Lock MI (2018). Stereotactic body radiation therapy for hepatocellular carcinoma: current trends and controversies. Technol Cancer Res Treat.

[59] Kimura T, Takahashi S, Takahashi I (2015). The time course of dynamic computed tomographic appearance of radiation injury to the cirrhotic liver following stereotactic body radiation therapy for hepatocellular carcinoma. PLoS One.

[60] Kim J, Jung Y (2017). Radiation-induced liver disease: current understanding and future perspectives. Exp Mol Med.

[61] Kimura T, Takahashi S, Kenjo M (2013). Dynamic computed tomography appearance of tumor response after stereotactic body radiation therapy for hepatocellular carcinoma: how should we evaluate treatment effects?. Hepatol Res.

[62] Lo SS, Dawson LA, Kim EY (2010). Stereotactic body radiation therapy for hepatocellular carcinoma. Discov Med.

[63] Liang SX, Zhu XD, Xu ZY (2006). Radiation-induced liver disease in three-dimensional conformal radiation therapy for primary liver carcinoma: the risk factors and hepatic radiation tolerance. Int J Radiat Oncol Biol Phys.

[64] Benson R, Madan R, Kilambi R, Chander S (2016). Radiation induced liver disease: a clinical update. J Egypt Natl Canc Inst.

[65] Hamami ME, Poeppel TD, Muller S (2009). SPECT/CT with 99mTc-MAA in radioembolization with 90Y microspheres in patients with hepatocellular cancer. J Nucl Med.

[66] Salem R, Parikh P, Atassi B (2008). Incidence of radiation pneumonitis after hepatic intra-arterial radiotherapy with yttrium-90 microspheres assuming uniform lung distribution. Am J Clin Oncol.

[67] Spina JC, Hume I, Pelaez A, Peralta O, Quadrelli M, Garcia Monaco R (2019). Expected and unexpected imaging findings after (90) Y transarterial radioembolization for liver tumors. Radiographics.

[68] Hamoui N, Minocha J, Memon K (2013). Prophylactic embolization of the gastroduodenal and right gastric arteries is not routinely necessary before radioembolization with glass microspheres. J Vasc Interv Radiol.

[69] Kim YC, Kim YH, Uhm SH (2010). Radiation safety issues in y-90 microsphere selective hepatic radioembolization therapy: possible radiation exposure from the patients. Nucl Med Mol Imaging.

[70] Kennedy AS, Nutting C, Coldwell D, Gaiser J, Drachenberg C (2004). Pathologic response and microdosimetry of (90) Y microspheres in man: review of four explanted whole livers. Int J Radiat Oncol Biol Phys.

[71] Sangro B, Inarrairaegui M, Bilbao JI (2012). Radioembolization for hepatocellular carcinoma. J Hepatol.

[72] Gil-Alzugaray B, Chopitea A, Inarrairaegui M (2013). Prognostic factors and prevention of radioembolization-induced liver disease. Hepatology.

[73] Sangro B, Gil-Alzugaray B, Rodriguez J (2008). Liver disease induced by radioembolization of liver tumors: description and possible risk factors. Cancer.

[74] Wahl DR, Stenmark MH, Tao Y (2016). Outcomes after stereotactic body radiotherapy or radiofrequency ablation for hepatocellular carcinoma. J Clin Oncol.

[75] Yoon SM, Lim YS, Park MJ (2013). Stereotactic body radiation therapy as an alternative treatment for small hepatocellular carcinoma. PLoS One.

[76] Sapisochin G, Barry A, Doherty M (2017). Stereotactic body radiotherapy vs. TACE or RFA as a bridge to transplant in patients with hepatocellular carcinoma An intention-to-treat analysis. J Hepatol.

[77] Miften M, Vinogradskiy Y, Moiseenko V et al (2018) Radiation dose-volume effects for liver SBRT. Int J Radiat Oncol Biol Phys. 10.1016/j.ijrobp.2017.12.290 10.1016/j.ijrobp.2017.12.290 PMC6095822 29482870

[78] Bujold A, Massey CA, Kim JJ (2013). Sequential phase I and II trials of stereotactic body radiotherapy for locally advanced hepatocellular carcinoma. J Clin Oncol.

[79] Salem R, Gordon AC, Mouli S (2016). Y90 radioembolization significantly prolongs time to progression compared with chemoembolization in patients with hepatocellular carcinoma. Gastroenterology.

[80] Facciorusso A, Serviddio G, Muscatiello N (2016). Transarterial radioembolization vs chemoembolization for hepatocarcinoma patients: a systematic review and meta-analysis. World J Hepatol.

[81] Parikh ND, Waljee AK, Singal AG (2015). Downstaging hepatocellular carcinoma: a systematic review and pooled analysis. Liver Transpl.

[82] Salem R, Gabr A, Riaz A (2018). Institutional decision to adopt Y90 as primary treatment for hepatocellular carcinoma informed by a 1,000-patient 15-year experience. Hepatology.

[83] Atassi B, Bangash AK, Bahrani A (2008). Multimodality imaging following 90Y radioembolization: a comprehensive review and pictorial essay. Radiographics.

[84] Cerny M, Chernyak V, Olivie D et al (2018) LI-RADS version 2018 ancillary features at MRI. Radiographics. 10.1148/rg.2018180052:180052 10.1148/rg.2018180052 30289735

[85] Cerny M, Bergeron C, Billiard JS (2018). LI-RADS for MR imaging diagnosis of hepatocellular carcinoma: performance of major and ancillary features. Radiology.

